# Strengthening North Pacific Influences on United States Temperature Variability

**DOI:** 10.1038/s41598-017-00175-y

**Published:** 2017-03-09

**Authors:** Justin A. Schulte, Sukyoung Lee

**Affiliations:** 1 Stevens Institute of Technology, Davidson Laboratory, Hoboken, NJ 07030 USA; 2 The Pennsylvania State University, University Park, Pennsylvania, PA 16823 USA

## Abstract

Changes in the frequency of occurrence of atmospheric circulation patterns under a changing climate system has important implications for regional climate variability. While many studies have focused on understanding the trends in the atmospheric pattern amplitudes and probabilities of occurrence, little attention has been given to how the linkages between climate variables and the large-scale patterns have been changing. Here we first document the strengthening relationship between an important North Pacific teleconnection - the East Pacific/North Pacific (EP/NP) pattern – and United States (US) temperature variability since the 1950s. The EP/NP pattern is linked to tropical convection, and consistently the coherence between US temperature anomalies and tropical convection anomalies has also been increasing during the same time period. This upward trend in convection-temperature alignment is most notable during autumn and winter and is absent during the summer. The relationship strengths and trends further suggest that the EP/NP pattern should be incorporated into monthly and seasonal outlooks that are of broad importance to agriculture, industry, and fisheries.

## Introduction

It is well-documented that the average global temperature is increasing but the trends in air temperature are not spatially uniform. The spatial heterogeneity of the trend patterns have motivated scientific investigators to understand the role of atmospheric dynamical mechanisms in creating the non-uniformities. One prevailing hypothesis^1–12^ is that a warming Arctic and Arctic sea ice loss impact mid-latitude weather patterns. However, despite the rapid sea ice loss, some studies^13, 14^ suggest that there are no robust trends in, for example, atmospheric blocking patterns, important atmospheric patterns related to droughts and temperature extremes. At least in the context of climate models, a difficulty in linking sea ice loss to atmospheric pattern changes is the large internal variability of the Earth’s climate system^15, 16^ compared to the forced response from sea ice loss^5^ and the small number of years with large sea ice loss^8^.

A competing hypothesis to the Arctic-weather linkage one is that recent prominent atmospheric circulation and associated climate anomalies across North America were related to tropical and North Pacific SSTs^17–20^ and that a wavier jet stream is the cause of Artic ice decline rather than the response to it^21–24^. The studies suggest that tropical SSTs may be useful for skillful seasonal temperature predictions. It is also possible that tropical and Arctic effects are not independent of each other because certain tropical convection anomalies can act to warm the Arctic^21–24^.

Despite the studies showing tropical and North Pacific linkages to US temperature, the Arctic Oscillation (AO, refs 25 and 26) index still remains one of the most widely used metrics to predict extratropical temperature variability because extreme values of the AO index are preceded by sudden stratospheric warming events with appreciable lead times^27, 28^. The underlying assumption for such a seasonal forecasting approach is that the AO explains appreciably more US temperature variability than any other existing mode of climate variability.

Regardless of the cause of recent abnormal atmospheric regimes, there is evidence supporting the notion that there are trends in the frequency of occurrence of large-scale circulation patterns^29^. An atmospheric circulation pattern that has received particular attention is the North American ridge-trough dipole and the Alaskan ridge^20, 30–33^, which have been identified as key circulation patterns related to the extreme eastern US winters of 2014 and 2015 and the California drought. There is some evidence that the amplitude of the Alaskan ridge pattern has been increasing^32^ and such amplification may be the result of the projection of anthropogenic forcing onto the pattern^30^. While numerous studies have examined ridge-trough dipole amplitude changes, little attention has been given to understanding how relationship strengths between the pattern and climate parameters have been changing. Such changes are important to identify and understand because of the implications for weather and seasonal prediction. Here, we show that the relationship between US temperature anomalies and the Eastern Pacific/North Pacific (EP/NP) atmospheric pattern has been increasing since the 1950’s. We also show that the EP/NP pattern is strongly correlated to the ridge-trough dipole so that the recently studied ridge-trough dipole is part of a hemispheric scale teleconnection pattern. Additionally, using a standard correlation analysis, we will test the hypothesis that the EP/NP index can explain, in many portions of the US, more temperature variability than the commonly used AO index in multiple seasons. We show that the upward trends in EP/NP-temperature relationship strengths has resulted in a greater number of US climate divisions for which the EP/NP pattern dominates the temperature variability.

## US Temperature and Atmospheric fields

The monthly US historical climate divisional data^34^ are used in this study (see Supplementary Figure S1). The observed data are quality controlled, extend back to the late 1800s, and are updated regularly. For this study, the period spanning 1950 to 2015 is used. The data are preferred to temperature reanalysis data sets because surface temperature from reanalysis products are inferred and do not necessarily use data from stations^35, 36^. Moreover, the divisional data are preferred to the daily station-based Global Historical Climate Network data set^37^ because station-based data are often nosier due to local climatological effects, which would impede the extraction of relevant climate signals. For all the time series, annual cycles were removed by subtracting the 1950 to 2015 (or 1980 to 2015) mean for each month from the monthly values of the same month. An additional linear detrending process was also performed to avoid spuriously large correlation coefficients between otherwise unrelated time series. The detrending step involved the computation of a least-square fit of a straight line to the data and the subsequent subtraction of the resulting function from the data. The step was also necessary for the scale-averaged coherence analysis (see Methods and Supplementary Material).

**Figure 1:**
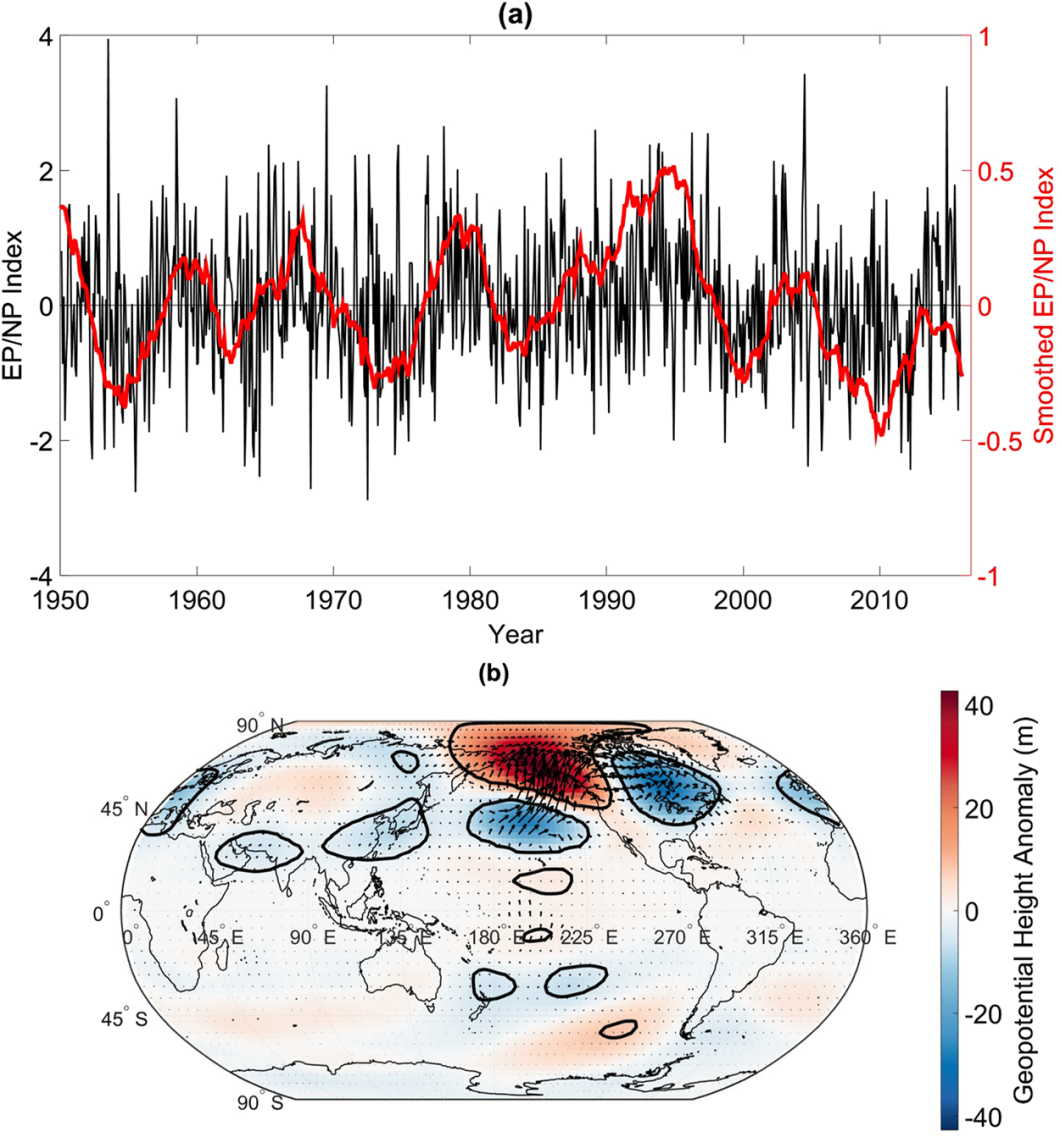
EP/NP Time series and EP/NP pattern. (**a**) The EP/NP index from 1950 to 2015 (black curve) together with the 5-year running mean. To reduce edge effects, the running mean was calculated by padding the left edge of the time series with the first value of the time series and padding the right end of the time series with the last value of the time series. Note that the raw and smoothed time series are plotted on different scales. (**b**) Composite of 500-hPa geopotential height anomalies for the positive EP/NP phase. Arrows indicate the wave activity flux (*m* ^2^ *s* ^2^), which have been scaled relative to maximum wave activity flux vector length of 0.89 *m* ^2^ *s* ^2^. The map was generated using MATLAB’s geoshow routine (MATLAB 2015a, http://www.mathworks.com/products/matlab/).

The intensity of the EP/NP pattern^38^ is measured using the EP/NP index (Fig. 1a) whose data from 1950 to 2015 were obtained from the National Oceanic Atmospheric Administration’s Earth System Research Laboratory (ESRL) website (ref. 38, http://www.esrl.noaa.gov/psd/data/climateindices/list/). The index was calculated using a rotated empirical orthogonal analysis of 500-hPa geopotential height. The December index, as computed by ERSL, does not exist ostensibly because the pattern is not a dominant one in December. However, US climate divisional December temperature variability was found to be associated with an EP/NP-like upper-atmospheric pattern (see Supplementary Figure S2). The December index used in the present analysis was constructed by filling the missing December values based on their relationships with 300-hPa streamfunction anomalies over Alaska (see Supplementary Materials). The pattern consists of three main anomaly centers, one over Alaska and two of similar sign over the central North Pacific Ocean and eastern US (Fig. 1b). In other words, a strong positive phase is associated with an amplified jetstream configuration in which a ridge is situated over Alaska and a trough is situated over the eastern US. The pattern also consists of tropical anomaly centers but they are weaker than the mid-latitude centers of action^39^. The formation of the anomaly centers are preceded by two wave trains that propagate from East Asia, the wave trains related to tropical convection^39^. Once the pattern is established, the pattern can be amplified by high-frequency eddies^39^. The wave activity flux vectors^40^ show poleward and eastward propagation, indicating that the wave trains associated with the positive EP/NP phase emanate from the North Pacific and result from anomalous forcing over the tropics and subtropics. The wave activity vectors show no evidence of polar influence on the anomalous wave pattern.

**Figure 2:**
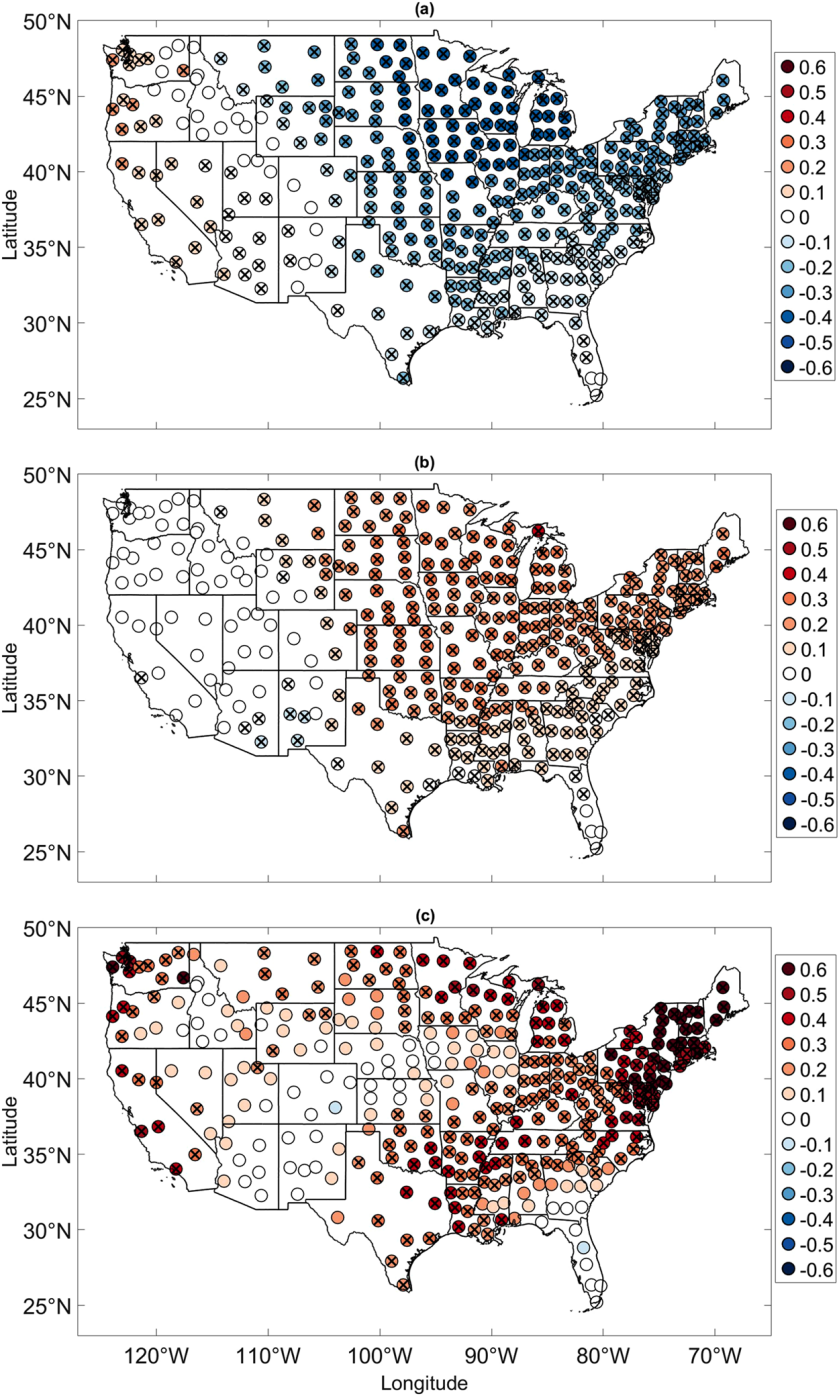
Strength and Trends in EP/NP index-temperature relationships. (**a**) Correlation between EP/NP index and temperature for each climate division from 1950 to 2015. Correlation coefficients statistically significant at the 5% level are indicated by crosses. (**b**) Linear trends per year in explained variance calculated using the sliding correlation analysis. Crosses denote those trends that are statistically significant at the 5% level. (**c**) Same as (**b**) except for the explained variance calculated using the scale-averaged coherence analysis. In this case, explained variance is defined as the scale-averaged coherence multiplied by 100 because coherence by definition is the fraction of variance of one time series explained by another time series. The map was generated using MATLAB’s geoshow routine (MATLAB 2015a, http://www.mathworks.com/products/matlab/).

In the current study, we focus on the EP/NP index. The selection of this index for the analysis as opposed to indices for the more well-known AO^25, 26^, North Atlantic Oscillation^41, 42^, Pacific Decadal Oscillation (PDO, refs 42 and 43), and Pacific-North American Teleconnection (PNA, ref. 44) pattern was statistically and physical motivated. The EP/NP pattern, though usually not thought of as a dominant pattern, was found to be most strongly related with monthly detrended temperature anomalies across the largest area of the US (Fig. 2a). More specifically, for about 70% of the US climate divisions, the EP/NP index was more strongly correlated with temperature than the AO index. The EP/NP index was found to be most strongly correlated with temperature in the fall and winter and the EP/NP-temperature relationships were found to be stronger than the AO-temperature relationships across the northern US (see Supplementary Figures S3–S7), the region impacted by the extreme winter of 2014–2015. It is also noted that relationships between indices for the PDO and the PNA and US temperature were generally found to be weaker than the EP/NP-temperature relationships (see Supplementary Figure S7). Additionally, the EP/NP pattern in the mid-latitudes resembles the ridge-trough dipole that has received recent attention^30^.

The 300-hPa streamfunction and convective precipitation fields are based on the National Center for Environmental Prediction (NCEP) reanalysis from 1950 to 2015^45^. The seasonal cycle was removed in a similar manner to the temperature data and the data were detrended at each grid point. The use of 300-hPa streamfunction is more suitable for the present analysis because geopotential height emphasizes high-latitude phenomena and underrepresents tropical phenomena. It is recognized that the NCEP precipitation data set may not be the most reliable precipitation product. Therefore, an additional precipitation data set is used to complement the analysis. The additional precipitation data set is based on the Climate Prediction Center (CPC) merged analysis of precipitation (CMAP; ref. 46) from 1979 to 2015. The data set consists of the merging of gauge observations, satellite estimates, and NCEP re-analyses. The SST analysis is based on the Kaplan SST Version 2 data set^47^ created by ERSL (ref. 48, http://www.esrl.noaa.gov/psd/data/gridded/data.kaplan_sst.html).

## Results

### US Temperature relationships with Atmospheric circulation patterns

The correlation pattern shown Fig. 2a indicates that the EP/NP index is most strongly correlated with temperature anomalies across the eastern two-thirds of the US, the relationships especially strong across northern portions. The negative correlation coefficients identified across the eastern US are a reflection of the eastern trough associated with the EP/NP positive phase. The results suggest that EP/NP index may be most useful in temperature prediction across the eastern US. Partitioning the analysis into the canonical winter (December–February), spring (March–May), summer (June–August), and fall (September–November seasons (see Supplementary Figures S3–S6) showed that the EP/NP index was correlated with US temperature during all seasons, the relationships particularly strong in the winter and fall. For some climate divisions, the magnitude of the correlation coefficients exceeded 0.7. The seasonal analysis suggests that the EP/NP pattern is important for prediction in all seasons. The AO index was also correlated with US temperature across the same regions (See Supplementary Figures S3–S7) but the strongest relationships were confined mainly to the southern US. The general pattern of EP/NP dominance in the north and AO dominance in south was generally consistent for all seasons (see Supplementary Figures S3–S6). The result suggests that the AO index offers more temperature predictability for southern portions of the US and the EP/NP offers more predictability for northern portions. Although the geographical distributions of the AO- and EP/NP-temperature correlations share resemblance, the temporal correlation between the two indices was found to be very weak (*r  *=−0.2).

**Figure 3:**
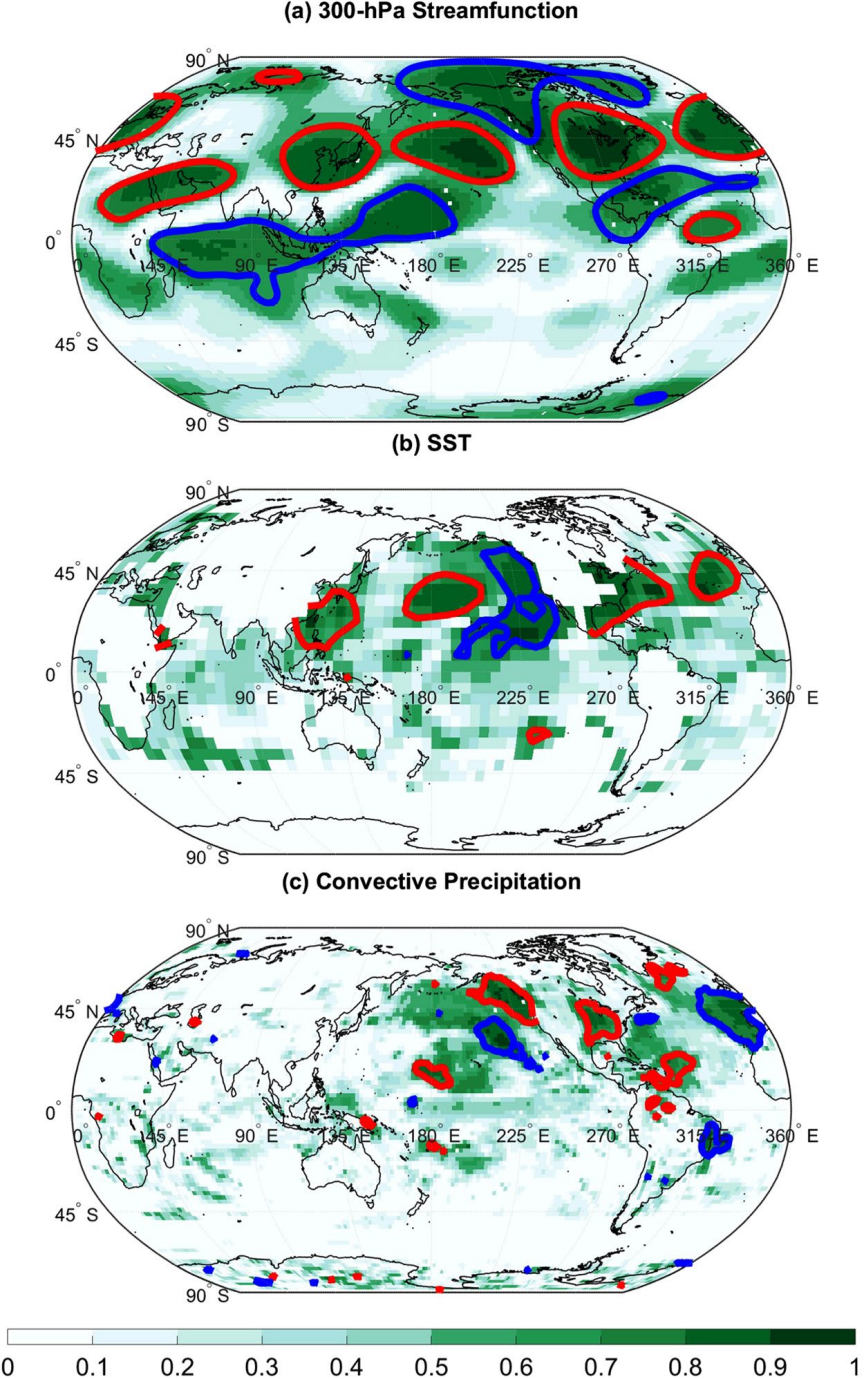
Correlation with 300-hPa streamfunction and Convective Precipitation. (**a**) The fraction of statistically significant correlation coefficients calculated between 300-hPa streamfunction and mean monthly temperature anomalies for the 344 US climate divisions. Red contours enclose regions where the mean correlation coefficient exceeds 0.1 and the blue contours enclose regions where the mean correlation coefficient falls below −0.1 Only climate divisions whose temperature time series was negatively correlated with the EP/NP index with at least 5% statistical significance were used in the computation of the means. (**b**) Same as (**a**) but for SST. (**c**) Same as (**a**) but for convective precipitation. The map was generated using MATLAB’s geoshow routine (MATLAB 2015a, http://www.mathworks.com/products/matlab/).

**Figure 4:**
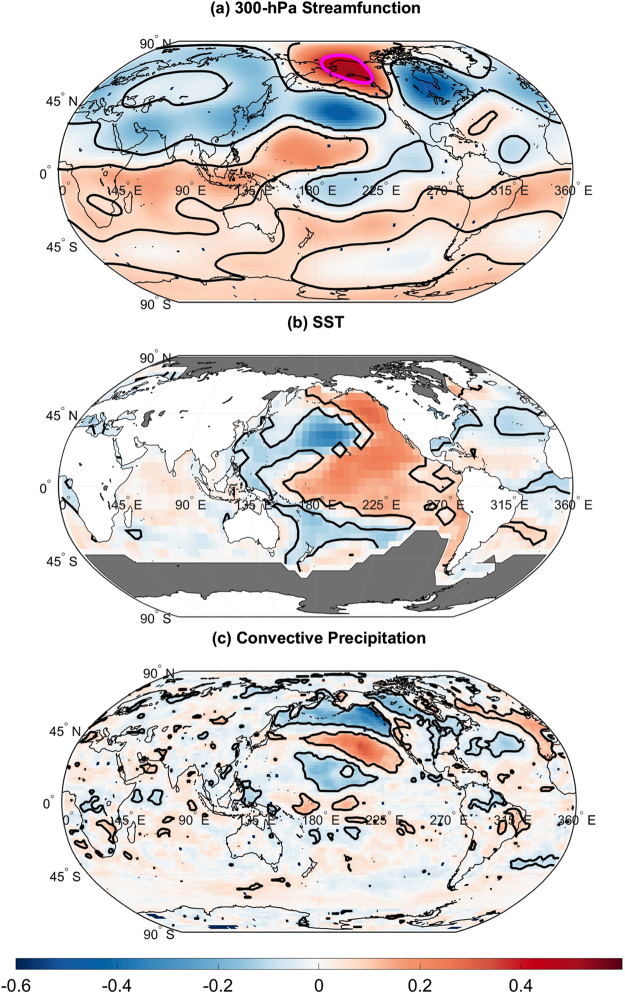
Correlation between EP/NP index and Atmospheric fields. (**a**) Correlation between EP/NP index and 300-hPa streamfunction. Black contours enclose regions of 5% statistically significance and the magenta contour encloses a region containing correlation coefficients greater than 0.4 used to fill the missing EP/NP index data. (**b**) Same as (**a**) but for SST. (**c**) Same as (**a**) but for convective precipitation. The map was generated using MATLAB’s geoshow routine (MATLAB 2015a, http://www.mathworks.com/products/matlab/).

We next examine large-scale circulation patterns associated with the divisional temperatures by computing correlation between each detrended climate divisional temperature time series and detrended 300-hPa streamfunction anomalies. To avoid having to define regions for spatial averaging, an additional data processing step, we plotted the fraction of climate divisional temperature time series that are significantly correlated with detrended 300-hPa streamfunction anomalies at each grid point (Fig. 3a). The mean correlation coefficient at each grid point was also computed to better illustrate the upper-atmospheric Rossby wave pattern. Only those time series that were negatively correlated with the EP/NP index were used in the calculation of the mean correlation coefficient to avoid averaging negative and positive relationships together and to ensure that resulting patterns for streamfunction, convective precipitation, and SST are produced from averaging correlation coefficients associated with the same climate divisions. The results indicate that the climate divisions with EP/NP-temperature anti-correlations are associated with a large-scale circulation pattern in which negative 300-hPa streamfunction anomalies are located over Alaska and the western tropical Pacific and positive 300-hPa streamfunction anomalies over the central North Pacific and eastern North America. The converse is true for the climate divisions whose temperature time series are positively correlated with the EP/NP index. A comparison of Fig. 3a with Fig. 4a shows that the pattern of mean correlation coefficients also resembles the correlation pattern obtained by correlating the EP/NP index with 300-hPa streamfunction, except the sign of the correlation coefficients are opposite. The correlation pattern across Alaska and the eastern US shown in Fig. 3a suggests that US temperature negative anomalies are primarily associated with a ridge over Alaska and a trough located over the eastern US, the so-called ridge-trough dipole^30, 31^.

A seasonal correlation analysis showed that the EP/NP-ridge-trough dipole relationship was strongest in the fall, spring, and winter (see Supplementary Figure S8), especially in the 1985 to 2015 period (see Supplementary Figure S9). The results indicate that the EP/NP pattern, like the ridge-trough dipole^30, 31^, was a dominant circulation pattern across the US during the winter but the EP/NP pattern appears to be also dominant in the fall and spring.

A similar analysis was conducted with detrended SSTs. The analysis revealed that nearly all US temperature anomalies are associated with an SST tripole such that the US temperature anomalies are associated with central Pacific, eastern Pacific, and western Pacific SST anomalies. For the US climate divisions negatively correlated with the EP/NP index, there was a positive relationship with both western and eastern Pacific SST anomalies and a negative relationship with central North Pacific SST anomalies. The pattern shown in Fig. 3b is similar to the SST pattern associated with the EP/NP index (Fig. 4b) and strongly resembles the previously identified Pacific Extreme Pattern (PEP) that is associated with summer hot day frequencies and eastward-propagating wave activity fluxes that converge over the eastern US^19^. Although the PEP pattern was only shown to be useful for summertime temperature prediction, the pattern was found to be also related to late winter (January–February) US temperature anomalies (see Supplementary Figure S10). The wintertime SST pattern found in this study resembles a previously identified NorthPacific Mode^17^ that was linked to the 2013–2014 winter temperature extremes.

Conducting a similar analysis with convective precipitation (Fig. 3c) revealed that about 50 to 70% of the climate division temperature time series are significantly correlated with convective precipitation across the western and central Pacific. The correlation analysis using the CMAP precipitation data set generally resulted in similar results, though fewer significant results were found across the western Pacific (see Supplementary Figure S11). It is also noted that the temperature time series are correlated with convective precipitation anomalies across western North American, which is expected because the Alaskan Ridge and the ridge-trough dipole were found to be related to California drought^20^, eastern US temperature anomalies^18^, and the EP/NP pattern as shown here.

The EP/NP index was correlated with convective precipitation (Fig. 4c) but no robust relationships were identified across the tropics. However, relationships were found to be slightly stronger for the analysis using the CMAP data (see Supplementary Figure S12). Recognizing that the EP/NP pattern is more frequently excited by tropical convection in the late winter and March^39^, we correlated the late winter and spring EP/NP index with late winter and spring convective precipitation but only spring relationships were identified (see Supplementary Figure S13) for the three selected periods 1950 to 1985, 1979 to 2015, and 1995 to 2015. The analysis produced more robust relationships, particularly for the 1979 to 2015 and 1995 to 2015 periods. The positive spring EP/NP index associated with enhanced convection over the central Pacific and decreased tropical convection over the maritime continent. The results further support the idea that EP/NP pattern is excited by tropical convection in the central and western Pacific^39^ but the importance of the tropics on the EP/NP may be confined seasonally.

### Trends in the US Temperature relationships with EP/NP Index

**Figure 5:**
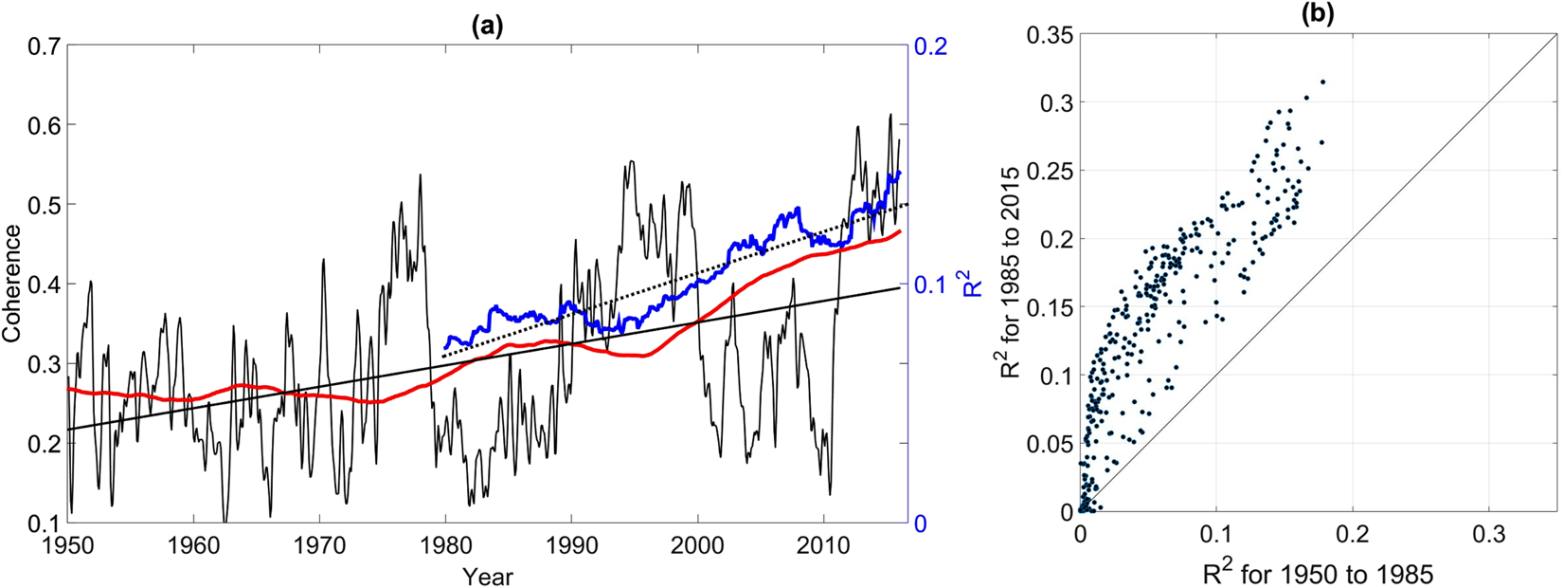
EP/NP Index Correlation and Coherence with Temperature. (**a**) Mean 30-year sliding squared correlation coefficient time series (blue curve) averaged across all 344 climate divisions for which statistically significant EP/NP-temperature relationship were identified in each 30-year segment. The scale-averaged wavelet coherence averaged across all US climate divisions is represented by the black curve. The stippled line represents the trend in coherence from 1980 to 2015 and the dotted line represents the trend from 1950 to 2015. Thick red line is the 30-year running mean of the coherence time series. Coherence and the mean 30-year sliding squared correlation coefficient time series are plotted on different scales. (**b**) *R* ^2^-values in the 1985 to 2015 segment plotted as a function of the *R* ^2^-values in the 1950 to 1980 segment. Points falling on the diagonal line represents those climate divisions for which no difference was found.

Figure 5a shows the 30-year sliding squared correlation coefficient (coefficient of determination) time series (referred to as the *R* ^2^ time series, hereafter) averaged across all 344 climate divisions plotted using the convention that the time series is plotted at the end year beginning in 1980^30^. Only statistically significant *R* ^2^ values were used in the averaging process. A clear upward trend in the *R* ^2^-values was identified and the EP/NP-temperature relationships were primarily strongest after 2010. The average *R* ^2^ values were found to have increased from 0.06 to 0.16 so that in the 1950–1980 time period the EP/NP index only explained an average of 6% for each climate division, compared to an average of nearly 16% of explained variance in the 1985 to 2015 time period. However, the positive trends in the *R* ^2^ values depended on the climate division in question, as shown in Fig. 5b. For many climate divisions, there was a two-fold increase in the *R* ^2^ values, the EP/NP index having explained over 30% of the temperature variability during the 1985–2015 period in some cases compared to only 15% during the 1950 to 1980 period. It also appears that the trends were largest for those temperature time series that were most strongly correlated with the EP/NP index. To check the sensitivity of the results to the detrending of the data, the analysis was repeated without detrending the temperature data and very similar findings were identified, enhancing confidence in the results from the detrended analysis. To ensure that the increase in correlation strengths from the 1950–1980 period to the 1985–2015 period were not related to the negative trend in the EP/NP index since the 1990s (see Fig. 1a), the sliding correlation analysis was performed by first detrending the EP/NP index in each individual 30-year segment before computing the correlation coefficients. The results were found to be similar, indicating that the identified trends were not strongly influenced by the negative EP/NP trend.

Trends were also identified when the analysis was partitioned into seasons (see Supplementary Figures S14 and S15). Positive trends were identified in all seasons except summer. The EP/NP-temperature relationships for the spring during the 1950 to 1985 period were found to be substantially weaker than those in the 1985 to 2015 period. No apparent trend was identified in the summer, the season for which the EP/NP temperature relationships were the weakest.

We also show the corresponding scale-averaged coherence (see Methods) time series, where scale-averaged coherence is a quantity bounded by 0 and 1. A value of 1 indicates the strongest possible relationship between two time series and 0 indicates the weakest possible relationship. Intuitively, it measures the fraction of variance of one time series explained by another at a particular moment of time. The scale-averaged time series were averaged across all climate divisions resulting in the time series shown in Fig. 5a. In agreement with the sliding correlation analysis, a mean upward trend was also identified in scale-averaged coherence. The coherence analysis also showed that higher-frequency fluctuations of coherence were superimposed on the background trend, the fluctuations in the coherence possibly related to the modulation of EP/NP-temperature relationships by another teleconnection pattern. The upward trend from 1980 to 2015 has been especially pronounced.

Figure 2b,c show the spatial distribution of the trends. The trends from the sliding correlation analysis and coherence analysis were found to be distributed similarly, with statistically significant upward trends, as indicated by black crosses, spanning the eastern two-thirds of the US. Both analyses identified the largest upward trends to be located across the northern and central portions of the US and the Northeast US. The result of the trends was an increase in the number of climate divisions for which the EP/NP pattern dominated temperature variability, particularly in the winter, spring, and fall seasons (see Supplementary Figure S16). The scale-averaged coherence analysis also identified relatively large trends across the Pacific Northwest, the region in which temperature is positively correlated with the EP/NP index. The coherence analysis results suggest that correlation coefficients have become more positive across the Pacific North West and more negative across the eastern two-thirds of the US.

### Trends in Temperature relationships with Circulation Patterns

**Figure 6:**
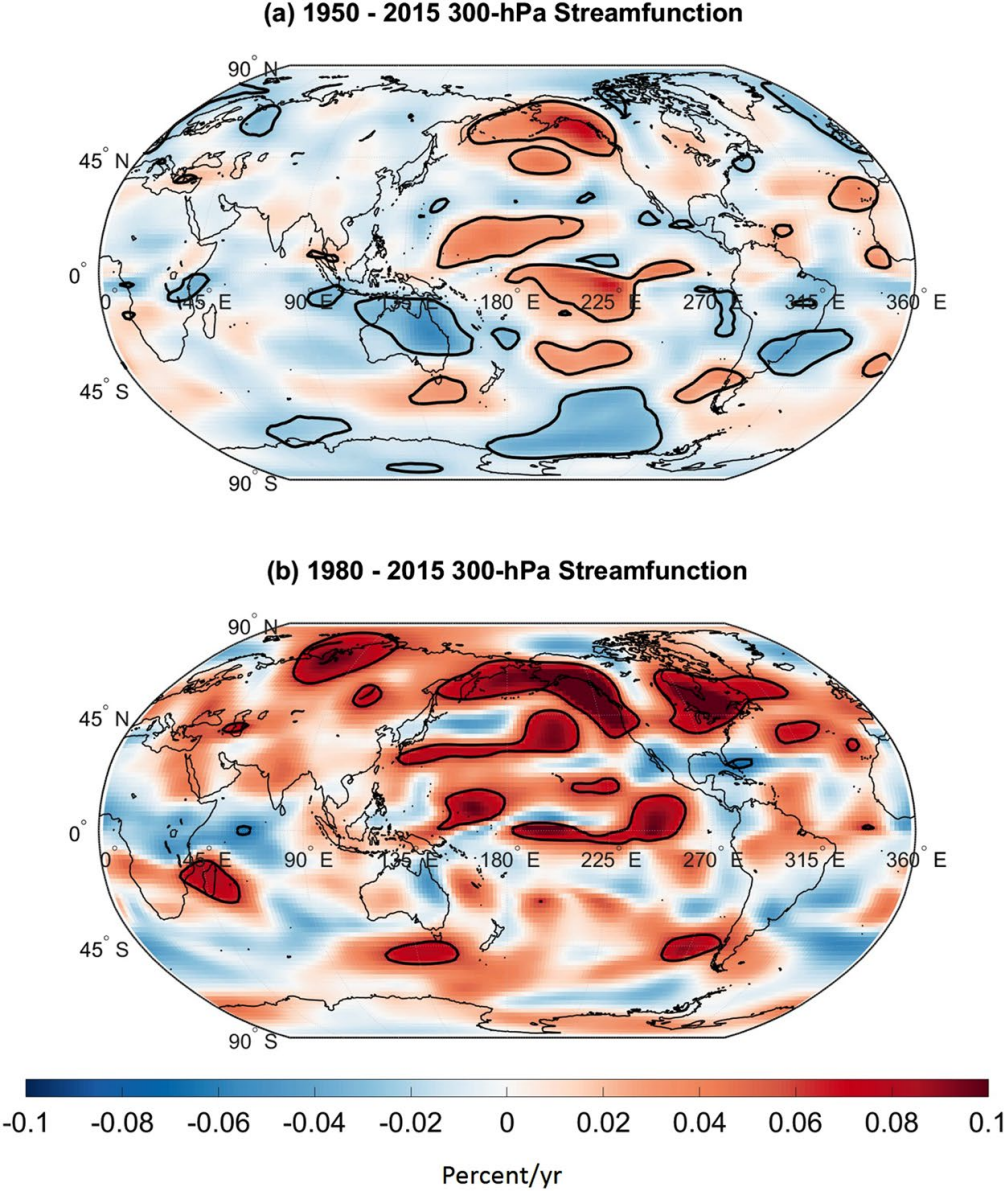
Trends in explained variance. (**a**) Average trend in explained variance calculated using scale-averaged coherence between temperature and 300-hPa streamfunction from 1950 to 2015. The average was computed by averaging the scale-averaged coherence trends associated with 17 US climate divisions whose *R* ^2^ trends exceeded the 95^th^ percentile of all trends calculated in the sliding correlation analysis. Black contours enclose regions of statistically significant trends. (**b**) Same as (**a**) but for 300-hPa streamfunction from 1980 to 2015. The map was generated using MATLAB’s geoshow routine (MATLAB 2015a, http://www.mathworks.com/products/matlab/).

Figure 6a shows the mean trend in scale-averaged coherence between temperature and 300-hPa streamfunction from 1950 to 2015. To cross-validate the scale coherence methodology, the mean of the coherence trends was computed using only the US climate divisions whose trends in the *R* ^2^ time series were greater than or equal to the 95^th^ percentile of all *R* ^2^ time series trends (see Supplementary Figure S17). It was found that there has been an upward trend in coherence between temperature and 300-hPa streamfunction anomalies across regions where the EP/NP index is significantly correlated with 300-hPa streamfunction. The results support the idea that the relationship between the EP/NP index and temperature has been strengthening. The largest upward trends were generally found across the central Pacific and across Alaska and so the trends in those regions contributed the largest to the trends in coherence shown in Fig. 5a. Consistent with Fig. 5a, the upward trends in coherence over Alaska and the central Pacific from 1980 to 2015 were larger than the 1950 to 2015 trends. A similar analysis conducted for precipitation from two data sets showed increasing trends in coherence with tropical precipitation (see Supplementary Figure S18) across the western and central Pacific, supporting the idea of a tropical influence.

## Discussion

The strong associations between US temperature variability and the EP/NP pattern together with the strengthening relationships suggest that a better understanding of the mechanisms governing the pattern’s evolution could improve predictability of US temperature from monthly to seasonal timescales.

Though spatially the EP/NP and AO-temperature correlation patterns were found to be similar, the EP/NP temperature relationships were found to be considerably stronger in northern portions of the US than the AO-temperature relationships and the AO-temperature relationships were found to be stronger in southern portions than the EP/NP-temperature relationships. The EP/NP index therefore is more useful for temperature prediction across northern portions. The inclusion of the EP/NP index is especially important recently given the EP/NP-temperature relationship trends that have resulted in an increased number of climate divisions for which the EP/NP dominates the temperature variability (see Supplementary Figure S19). Current wintertime seasonal forecasts use the snow advance index (SAI) to predict the wintertime AO and subsequently produce wintertime temperature outlooks for the US^49^. However, even though the SAI can skillfully forecast the wintertime AO, a strong EP/NP pattern could produce monthly temperature anomalies of opposing sign. This opposition is expected because the indices are uncorrelated so that a negative phase of one pattern need not occur with a positive phase of the other.

The strong EP/NP-US temperature relationships and upward trends in the relationship strengths also suggest that EP/NP pattern could provide a useful framework for understanding how long-term variations in climate conditions could render dynamical alterations of atmospheric circulation patterns. Increases in wave activity from the tropics and tropical atmospheric circulation anomalies associated with the EP/NP pattern (see Supplementary Figure S20) suggests a strengthening tropical influence on the EP/NP pattern. The positive trend in coherence identified in the present analysis therefore may have resulted from increasing tropical influences. The two-fold linkage between the EP/NP pattern and two possible anthropogenic sensitive components of the climate system raise a possibility that the identified trends may at least partially be a response to human activity.

One caveat of the present analysis is that the EP/NP-temperature correlation trends could have resulted from changes in the observing system and the inclusion of satellite data into reanalysis products. However, an inspection of Fig. 1b shows that the positive coherence began in the early 1970s before the satellite era. Additionally, the period after satellite inclusion in the 1980s was associated with relatively low coherence, casting some doubt that the strengthening in EP/NP coherence with temperature was related to changes in the observing system. While the decadal time-scale trends suggest a forced response in the climate system, one cannot exclude the possibility that such trends are related to internal climate dynamics, given the large background noise^15, 16^. The results from the present work highlight that teleconnection patterns that are regarded as being dominant in terms of fractional variance of geopotential height variability (such as the AO) are not necessarily the most influential circulation patterns for US temperature anomalies, and that to improve future predictability of US temperature anomalies, it is necessary to understand why tropical influences have been strengthening.

## Methods

### Correlation analysis

The relationships between the EP/NP index and US climate divisional data was first quantified using a sliding correlation analysis^30^. We broke the data from 1950 to 2015 into 30-yr segments and computed the Pearson correlation coefficient between mean monthly temperature anomalies and the monthly EP/NP index for each segment. The calculation was repeated for each of the 344 US climate divisions. Squaring the correlation coefficients resulted in a (coefficient of determination) *R* ^2^ time series for each of the 344 climate divisions (see Supplementary Figure S1), measuring how much temperature variability can be linearly predicted from the EP/NP index. The trends were based on *R* ^2^ values because wavelet coherence is bounded by 0 and 1 exactly like traditional *R* ^2^ values. The *R* ^2^ time series began at the last year of the first segment^30^.

The statistical significance of the linear trends in the *R* ^2^ time series was assessed using Monte Carlo methods because of the large autocorrelation found in the time series. The Monte Carlo experiment was performed as follows: first the lag-1 autocorrelation coefficients of the EP/NP index and the temperature time series were computed. Then, 1000 surrogate red-noise time series with same lengths and autocorrelation coefficients as the input time series were generated. For each pair of surrogate time series, the sliding correlation coefficient and the associated *R* ^2^ time series was computed. Computing the absolute value of the linear trend for each surrogate *R* ^2^ time series, resulted a null distribution of linear trend magnitudes. A linear trend was then deemed statistical significant at the 5% significance if it exceeded the 95^th^ percentile of the null distribution.

### Scale-averaged coherence analysis

To complement the sliding correlation analysis and to gain more confidence results, a wavelet coherence analysis^50, 51^ was conducted. The scale-averaged coherence analysis has numerous advantages over the sliding correlation analysis. The first advantage is that one does not need select an arbitrary window size, which was selected as 30 years in this study. A second benefit is that there are no edge effects (see Supplementary material), contrasting with the sliding correlation analysis in which information at the edges of the time series is lost. A third benefit is that there is no longer a need to arbitrarily selecting a start date of the time series, say 1980, in the sliding correlation analysis.

The wavelet transform of a time series *X* is given by

1 $${W}_{n}^{x}({\rm{s}})=\sqrt{\frac{2\delta t}{s}}\sum _{n^{\prime} =0}^{N-1}{x}_{{n}^{\text{'}}}{\psi }^{\ast }[\frac{(n^{\prime} -n)\delta t}{s}],$$

where $\psi$ is the Morlet wavelet given by

2 $$\psi (\eta )={\pi }^{-1/4}{e}^{i{\omega }_{0}\eta }{e}^{-\frac{1}{2}{\eta }^{2}},$$

${\omega }_{0}=6$ is the dimensionless frequency, *t* is time, *s* is the wavelet scale, $\delta t$ is a time step (1 month) determined from the data, *N* = 792 is the length of the time series, and $\eta =s\cdot t$ ^50, 52^. The asterisk in Eq. 1 denotes the complex conjugate. The wavelet transform was computed at a discrete set of scales $({s}_{j};j=0,\,1,\ldots ,\,J)$, with

3 $${s}_{j}={s}_{0}{2}^{j\delta },$$

4 $$J={\delta }^{-1}lo{g}_{2}(N\delta t/{s}_{0}),$$

and *δ* = 0.5.

The correlation between two time series was computed as a function of time using scale-averaged coherence. The scale-averaged coherence between a time series *X* and *Y* is given by

5 $${R}_{n}^{2}=\frac{{|\sum _{j=0}^{J}{W}_{n}^{xy}({s}_{j})|}^{2}}{(\sum _{j=0}^{J}{|{W}_{n}^{x}({s}_{j})|}^{2})(\sum _{j=0}^{J}{|{W}_{n}^{y}({s}_{j})|}^{2})},$$

where ${W}_{n}^{xy}$(*s*_*j*_) is the cross-wavelet transform between *X* and *Y*. The quantity is bounded by 0 and 1 by the Schwartz inequality. If ${R}_{n}^{2}$ = 1, then there is a perfect association between the two time series at the time index *n* and ${R}_{n}^{2}$ = 0 indicates no association between the time series. The temporal resolution of ${R}_{n}^{2}$ is approximately twice the decorrelation length of the Morlet wavelet at *s*_0_ = 2, which is 5.7 months in the present study.

The statistical significance of the trends for ${R}_{n}^{2}$ was also assessed using Monte Carlo methods. Red-noise time series with the same lag-1 autocorrelation coefficients and lengths as the input time series were generated 1,000 times. For each of the synthetic time series, ${R}_{n}^{2}$ was computed and a linear trend was fitted to the data using least-squares regression. The absolute value of the slopes was computed to create a null distribution of 1000 slope magnitudes. The 95^th^ percentile of the null distribution was used as the critical level of the test applied at the 5% significance level.

## Electronic supplementary material

Supplementary Information

## References

[1] Francis JA, Vavrus SJ (2012). Evidence linking Arctic amplification to extreme weather in mid-latitudes. Geophys. Res. Lett..

[2] Screen JA, Simmonds I (2013). Exploring links between Arctic amplification and mid-latitude weather. Geophys. Res. Lett..

[3] Screen JA (2013). Influence of Arctic sea ice on European summer precipitation. Environ. Res. Lett..

[4] Screen, J. A., Deser, C., Simmonds, I. & Tomas, R. The atmospheric response to three decades of observed Arctic sea ice loss. Clim. Dyn. (2013).

[5] Screen JA, Deser C, Simmonds I, Tomas R (2014). Atmospheric impacts of Arctic sea-ice loss, 1979–2009: separating forced change from atmospheric internal variability. Clim. Dyn..

[6] Tang Q, Zhang X, Francis JA (2014). Extreme summer weather in northern mid-latitudes linked to a vanishing cryosphere. Nature Clim. Change..

[7] Cohen J (2014). Recent Arctic amplification and extreme mid-latitude weather. Nat. Geosci..

[8] Vihma T (2014). Effects of Arctic sea ice decline on weather and climate. Surv. Geophys..

[9] Francis JA, Skific N (2015). Evidence linking rapid Arctic warming to mid-latitude weather patterns. Phil. Trans. R. Soc. A..

[10] Francis JA (2015). The Arctic matters: extreme weather responds to diminished Arctic sea ice. Environ. Res. Lett..

[11] Screen JA, Deser C, Sun L (2015). Projected changes in regional climate extremes arising from Arctic sea ice loss. Environ. Res. Lett..

[12] Screen JA, Deser C, Sun L (2015). Reduced risk of North American cold extremes due to continued Arctic sea ice loss. Bull. Amer. Meteorol. Soc..

[13] Barnes EA (2013). Revisiting the evidence linking Arctic amplification to extreme weather in midlatitudes. Geophys. Res. Lett..

[14] Barnes EA, Dunn-Sigouin E, Masato G, Woollings T (2014). Exploring recent trends in Northern Hemisphere blocking. Geophys. Res. Lett..

[15] Deser C, Phillips AS, Alexander MA, Smoliak BV (2014). Projecting North American climate over the next 50 years: uncertainty due to internal variability. J. Clim..

[16] Shepherd TG (2014). Atmospheric circulation as a source of uncertainty in climate change projections. Nat. Geosci..

[17] Hartmann DL (2015). Pacific Sea Surface Temperature and the Winter of 2014. Geophys. Res. Lett..

[18] Palmer T (2014). Record-breaking winters and global climate change. Science..

[19] McKinnonn KA, Rhines A, Tingley MP, Huybers P (2016). Long-lead predictions of eastern United States hot days from Pacific sea surface temperatures. Nat. Geosci..

[20] Seager R, Henderson N (2016). On the Role of Tropical Ocean Forcing of the Persistent North American West Coast Ridge of Winter 2013/14. J. Clim..

[21] Yoo C, Feldstein S, Lee S (2011). Impact of the Madden-Julian Oscillation (MJO) trend on the polar amplification of surface air temperature during 1979–2008 boreal winter. Geophys. Res. Lett..

[22] Lee S, Gong T, Johnson N, Feldstein SB, Pollard D (2011). On the possible link between tropical convection and the Northern Hemisphere Arctic surface air temperature change between 1958 and 2001. J. Clim..

[23] Lee S (2014). A theory for polar amplification from a general circulation perspective. Asia-Pacific J. Atmos. Sci..

[24] Ding Q (2014). Tropical forcing of the recent rapid Arctic warming in northeastern Canada and Greenland. Nature..

[25] Thompson DWJ, Wallace JM (1998). The Arctic Oscillation signature in the wintertime geopotential height and temperature fields. Geophys. Res. Lett..

[26] Thompson DWJ, Wallace JM (2001). Regional climate impacts of the Northern Hemisphere annular mode. Science..

[27] Baldwin MP, Dunkerton TJ (1999). Propagation of the Arctic Oscillation from the stratosphere to the troposphere. J. Geophys. Res..

[28] Baldwin MP, Dunkerton TJ (2001). Stratospheric harbingers of anomalous weather regimes. Science.

[29] Horton DE (2015). Contribution of changes in atmospheric circulation patterns to extreme temperature trends. Nature..

[30] Wang S-Y, Hipps L, Gillies RR, Yoon J-H (2014). Probable causes of the abnormal ridge accompanying the 2013–2014 California drought: ENSO precursor and anthropogenic warming footprint. Geos. Phys. Lett..

[31] Wang S, Huang W, Yoon J (2015). The North American winter “dipole” and extremes activity: A CMIP5 assessment. Atmos. Sci. Lett..

[32] Swain DL, Horton DE, Singh D, Diffenbaugh NS (2016). Trends in atmospheric patterns conducive to seasonal precipitation and temperature extremes in California. Science Advances..

[33] Singh D (2016). Recent amplification of the North American winter temperature dipole. J. Geophys. Res..

[34] Guttman NV, Quayle RG (1996). A historical perspective of US climate divisions. Bull. Amer. Meteor. Soc..

[35] Simmons A (2004). Comparison of trends and low-frequency variability in CRU, ERA-40, and NCEP/NCAR analyses of surface air temperature. J. Geophys. Res..

[36] Hofstra N, New M, McSweeney C (2010). The influence of interpolation and station network density on the distributions and trends of climate variables in gridded daily data. Clim. Dynam..

[37] Menne MJ, Durre I, Vose RS, Gleason BE, Houston TG (2012). An overview of the global historical climatology network-daily database. J. Atmos. Ocean. Technol..

[38] National Oceanic Atmospheric Administration/Earth System Research Laboratory Physical Sciences Division, Climate Indices: Monthly Atmospheric and Ocean Time Series (Date of access: 16/01/2016).

[39] Tan BK, Yuan JC, Dai Y, Feldstein SB (2015). The Linkage between the Eastern Pacific Teleconnection Pattern and Convective Heating over the Tropical Western Pacific. J. Clim..

[40] Takaya K, Nakamura H (2001). A formation of a phase-independent waveactivity flux for stationary and migratory quasigeostrophic eddies on a zonally varying basic flow. J. Atmos. Sci..

[41] Hurrell JW (1995). Decadal trends in the north Atlantic oscillation: regional temperatures and precipitation. Science..

[42] Hurrell JW, van Loon H (1997). Decadal variations in climate associated with the North Atlantic Oscillation Climatic Change..

[43] Mantua NJ, Hare SR, Zhang Y, Wallace JM, Francis RC (1997). A Pacific decadal climate oscillation with impacts on salmon. Bull. Amer. Met. Soc..

[44] Wallace JM, Gutzler DS (1981). Teleconnections in the Geopotential Height Field during Northern Hemisphere Winter. Mon. Weather Review..

[45] Kalnay ME (1996). The NCEP/NCAR Reanalysis Bull. Am. Meteorol. Soc..

[46] Xie P, Arkin PA (1997). Global precipitation: a 17-year monthly analysis based on gauge observations, satellite estimates, and numerical model outputs. Bull. Amer. Met. Soc..

[47] Kaplan A (1998). Analyses of global sea surface temperature 1856–1991. J. Geophys. Res..

[48] National Oceanic Atmospheric Administration/Earth System Research Laboratory Physical Sciences Division, Kaplan Extended SST V2 (Date of access: 29/10/2016).

[49] Cohen J, Jones J (2011). A new index for more accurate winter predictions. Geophys. Res. Lett..

[50] Grinsted A, Moore JC, Jevrejeva S (2014). Application of the cross wavelet transform and wavelet coherence to geophysical time series, Nonlin. Process. Geophys..

[51] Schulte JA, Najjar RG, Li M (2016). The influence of climate modes on streamflow in the Mid-Atlantic region of the United States J. Hydrology: Regional Studies..

[52] Torrence C, Compo GP (1998). A practical guide to meteorology. Bull. Amer. Met. Soc..

