# Supplementary material for: Strengthening North Pacific Influences on United States Temperature Variability
Source: Sci Rep. 2017 Mar 9;7:124. doi: 10.1038/s41598-017-00175-y (PMC5427841; doi:10.1038/s41598-017-00175-y)
Supplement: Supplementary file 1 — Supplementary Information [file 41598_2017_175_MOESM1_ESM.pdf]

Strengthening North Pacific Influences on United States Temperature Variability

Justin A. Schulte[1]

Sukyoung Lee[2]

1. Stevens Institute of Technology, Davidson Laboratory, Hoboken, NJ, 07030

2. The Pennsylvania State University, University Park, PA, 16823

Supplementary material

The selection of the climate index to use in the analysis was motivated by how well the climate index correlates with mean monthly temperature anomalies across the US. The AO index was found to be correlated with temperature across a large region of the US but the maximum correlation strength was only 0.38 (Figure S7). Approximately 20% of the climate divisions from temperature-EP/NP correlation analysis had correlation coefficient magnitudes exceeding 0.38. A similar result was found when correlating the PDO index with US temperature anomalies. The PNA-temperature correlation maps shows localized regions where the magnitude of the correlation coefficients exceeded 0.4. However, such regions are considerably smaller those found when correlating the EP/NP index with temperature (Figure 1c and 2a).

Evidence for a tropical connection to the EP/NP pattern was supported by correlating the EP/NP index with precipitation from two separate data products. The correlation analysis using the CMAP precipitation data set revealed statistically significant relationships between precipitation and the EP/NP index across the western and central Pacific (see Supplementary Figure S12). Using the same precipitation product, many US climate divisional temperature time series were found to be related with precipitation across the central Pacific and, to a lesser extent, across the western Pacific (Figure S12).

Trends in coherence between US temperature and precipitation was also computed (see Supplementary Figure S18). No significant trends in coherence were found from 1950 to 2015 across the central equatorial Pacific. That lack of significant coherence trends with tropical convection suggests that increasing coherence with the EP/NP index is related to another process. However, it is uncertain if the rather noisy results are related to the quality of the reanalysis data set, especially prior to 1979. Examining the 1980-2015 period in isolation, the region of significant upward trend is collocated with the region in which the EP/NP index is correlated with tropical convection and the region in which the majority of US climate division are related to convective precipitation. The coherence trends calculated using the CMAP data set (see Supplementary Figure S18) produced even more robust trends across the central and western Pacific and the colocation of the statistically significant EP/NP-precipitation relationships and the significant upward trends was even clearer. The results imply that not only has US temperature anomalies become more coherent with changes in the EP/NP pattern but also the tropical convection that excites the EP/NP pattern.

A major assumption of the wavelet analysis is that the data are equally spaced and continuous. Therefore, missing EP/NP index data in December were filled by correlating the index

with 300-hPa streamfunction anomalies and linearly regressing the index with a 300-hPa streamfunction anomaly time series calculated by averaging streamfunction in the region enclosed by a magenta contour shown in Figure 4a. The exact method by which the filling is done is not important because scale-averaged coherence at a point time is a weighted quantity based on data points surrounding the point of interest (see Methods). The simplest and most straightforward approach was thus adopted. Although the pattern is said not to be dominant one in December, there appears to be a large number of climate divisions whose December temperature time series is related to an upper-tropospheric circulation pattern (see Supplementary Figure S2) strongly resembling the EP/NP pattern (Figure 1). The result suggests that while the pattern may not be a dominant one from the perspective of an EOF analysis, the pattern seems to be a physically relevant one for the US.

Trends in time series can produce spuriously large correlation coefficients. We show that the same holds for scale-averaged coherence. To do so, two realizations, w_1 and w_2, of a Gaussian white noise process were generated. A synthetic trend was then added to the time series resulting in two new time series

$$T_1(t) = w_1(t) + 0.1t \qquad \text{(Eq. S1)}$$

and

$$T_2(t) = w_2(t) + 0.1t. \qquad \text{(Eq. S2)}$$

The scaled-averaged coherence was computed between T_1 and T_2, between $w_1$and w_2, and between T_1 and T_2 after detrending was performed. The results are presented in Supplementary Figure S21. The scale-averaged coherence between T_1 and T_2 (black line) is spuriously large at the edges of the time series and decreases toward the center of the time series. In contrast, the scale-averaged coherence between $w_1$and w_2 (magenta line) does not exhibit the high coherence at the edges and, in fact, if one detrends both T_1 and T_2 and then calculates the scale-averaged coherence (dotted line) one can remove the spuriously large coherence at the edges. It was found that the influence of the trend depends on how large the trends are in the input time series and the length of the time series (not shown). Shorter time series were found to need larger trends to produce the trend-induced edge effects. It is noted that the synthetic trend generated in the above experiment was quite exaggerated so that application of the coherence analysis to real data will generally produce considerably more subtle edge effects, perhaps too small to detect.

It is known that there are edge effects in wavelet analysis due to the finite length of the time series. The region in which edge effects are important is called the cone of influence (COI; 52). While effects are important in making inferences about wavelet power and cross-power spectra, they do not seem to interfere with the interpretation of scale-averaged coherence results. The lack of edge effects for scale-averaged coherence was demonstrated empirically using a simple experiment. In the experiment, two sinusoids of identical period (arbitrary units), length, and amplitude were generated. The scale-averaged cross-power and coherence was computed between them. Theoretically, the scale-averaged coherence should be unity at all times and the cross-power should be independent of time because the sinusoids are stationary and the proprieties of them are identical at all times. The experiment was computed for different periods of the sinusoids. The

results are presented in Supplementary Figure S22. As expected for the scale-averaged cross-power, the cross-power is reduced near the edges. The reduction in power is largest for the pair of sinusoids with period of 62. The result is consistent with how the COI becomes larger at higher periods. In contrast, for the scale-averaged coherence, no time dependence was found, with the scale-averaged coherence being unity at all times. Moreover, the results were not dependent on the period of the sinusoids used to calculate the scale-averaged coherence. The result implies that the scale-averaged coherence results near the edges of the time series can be meaningfully interpreted so that trends calculated are not artifacts of the edge effects.

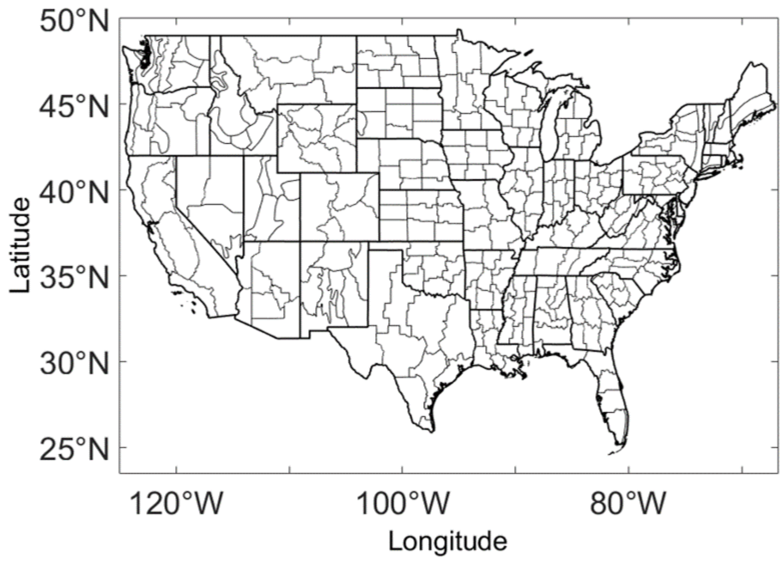

Figure S1. The locations of the 344 US climate divisions. The map was generated using MATLAB's geoshow routine (MATLAB 2015a, http://www.mathworks.com/products/matlab/).

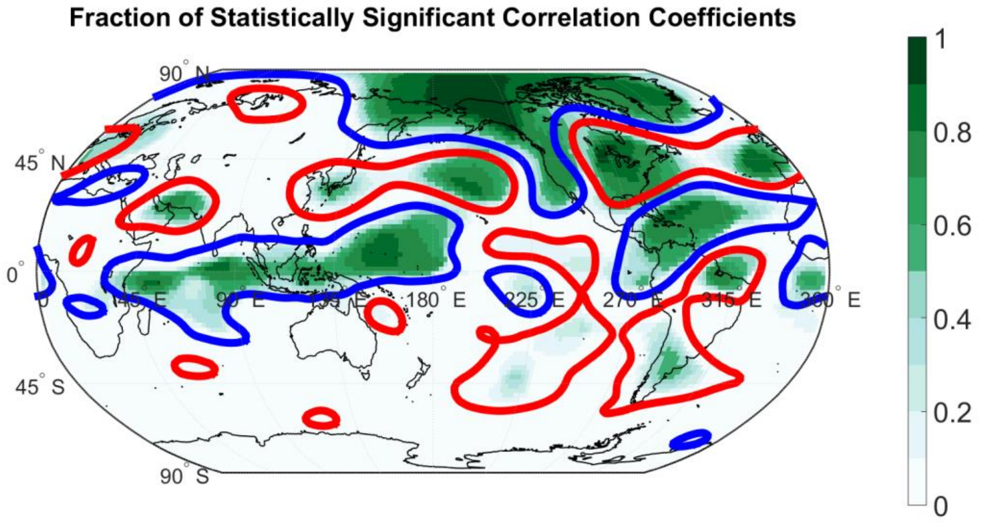

Figure S2. Fraction of US climate divisions for which detrended December temperature anomalies are significantly correlated at the 5% level with detrended 300-hPa streamfunction anomalies. Red contours enclose regions where the mean correlation coefficient was greater than 1.0 and the blue contours enclose regions where the mean correlation coefficient was less than -1.0. Only US climate divisions that were negatively correlated with the EP/NP index were used to compute the mean correlation coefficients. The map was generated using MATLAB's geoshow routine (MATLAB 2015a, http://www.mathworks.com/products/matlab/).

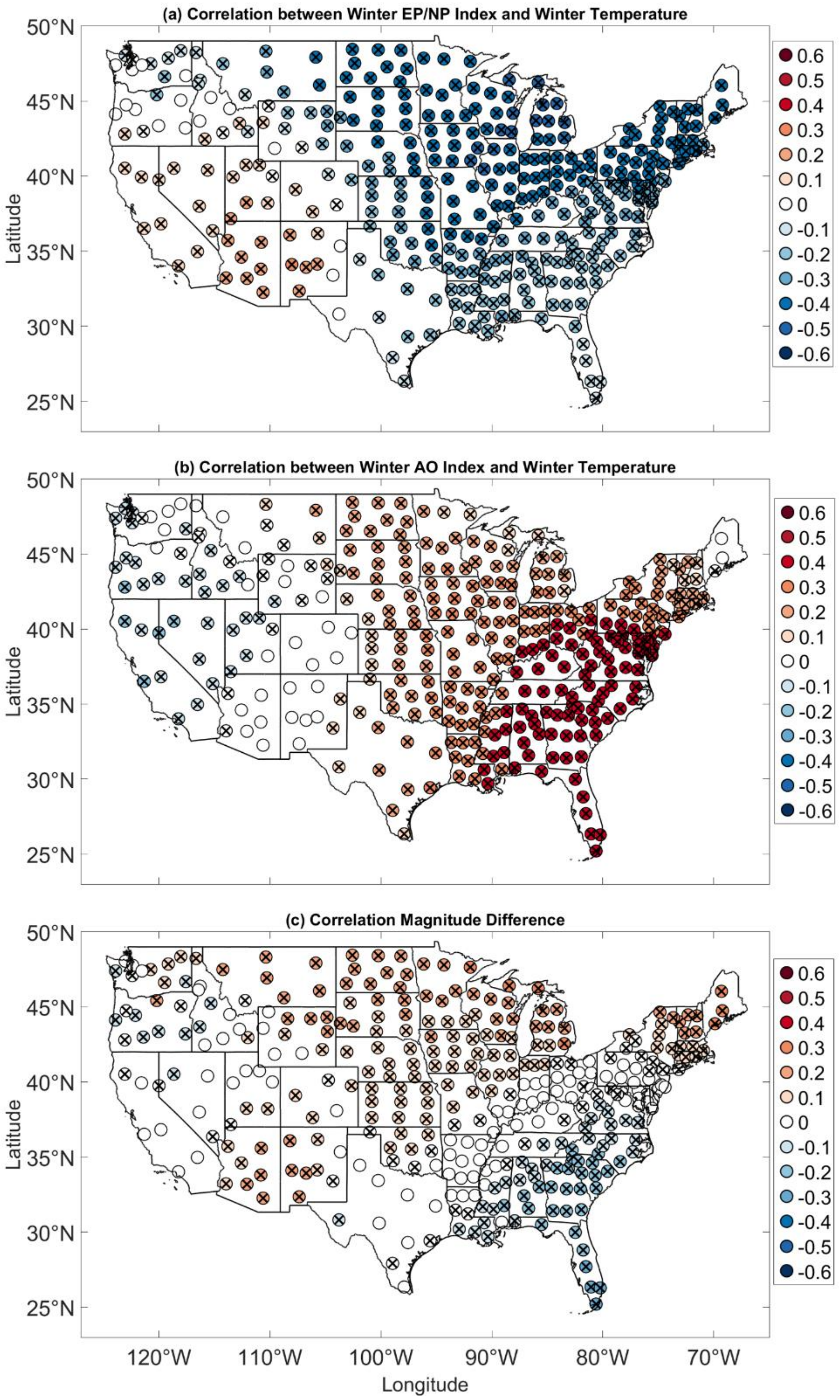

Figure S3. (a) Correlation between winter EP/NP index and Winter US climate divisional temperature. (b) Same as (a) but for the winter AO index. (c) Difference between the magnitude of the correlation coefficients in (a) and those in (b). Crosses denote those results that are statistically significant at the 5% level. The map was generated using MATLAB's geoshow routine (MATLAB 2015a, http://www.mathworks.com/products/matlab/).

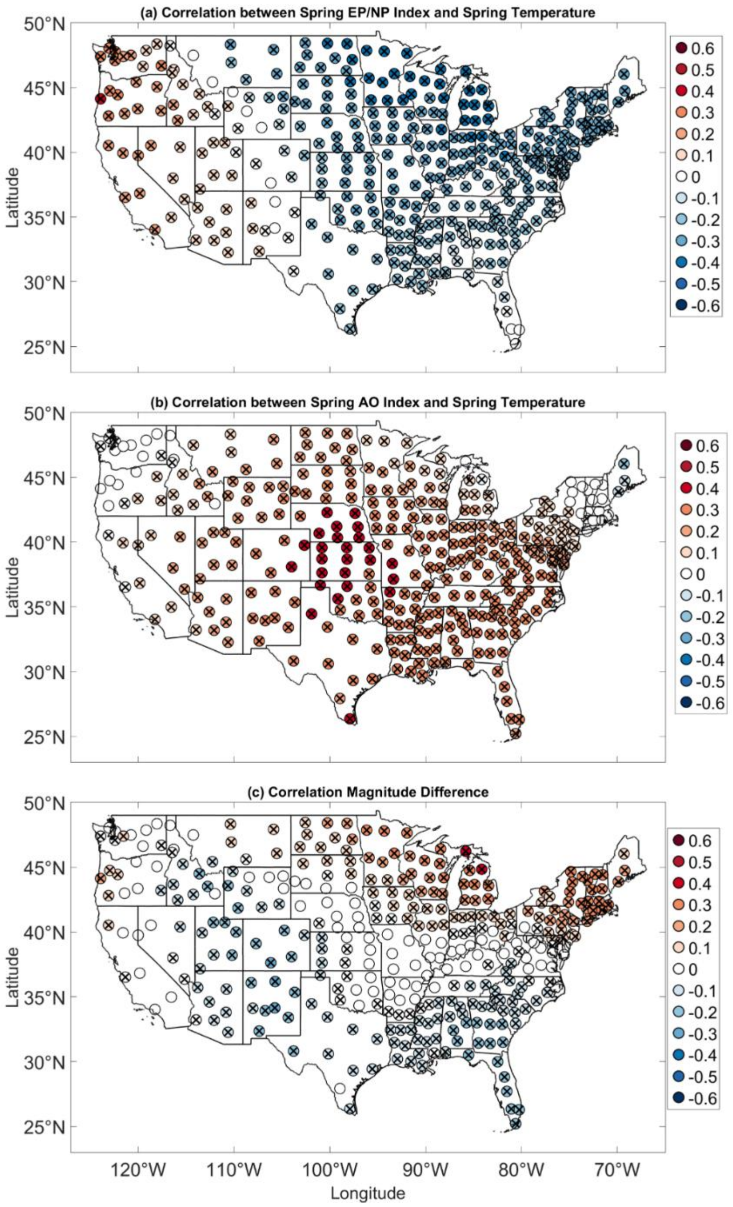

Figure S4. (a) Correlation between Spring EP/NP index and Spring US climate divisional temperature. (b) Same as (a) but for the winter AO index. (c) Difference between the magnitude of the correlation coefficients in (a) and those in (b). Crosses denote those results that are statistically significant at the 5% level. The map was generated using MATLAB's geoshow routine (MATLAB 2015a, http://www.mathworks.com/products/matlab/).

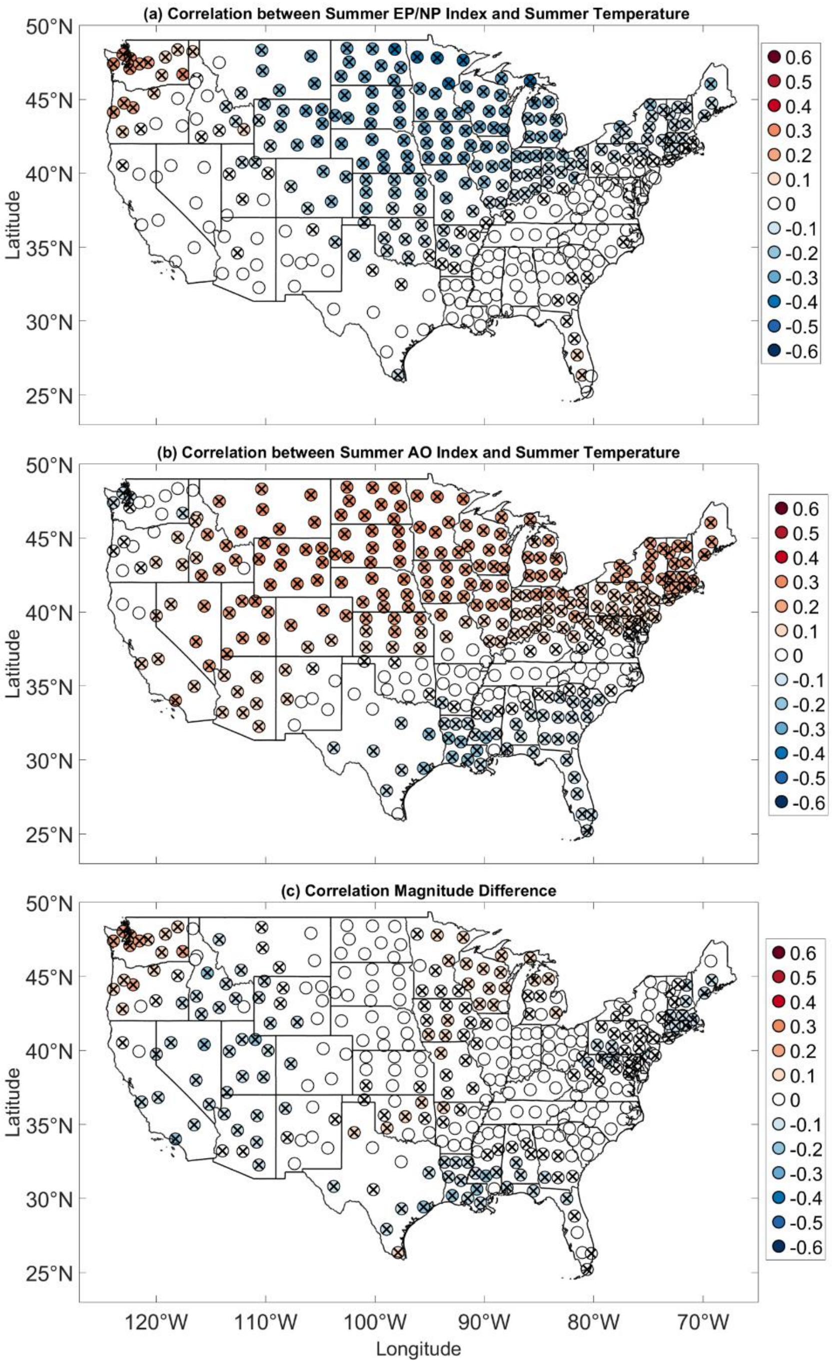

Figure S5. (a) Correlation between Summer EP/NP index and Summer US climate divisional temperature. (b) Same as (a) but for the summer AO index. (c) Difference between the magnitude of the correlation coefficients in (a) and those in (b). Crosses denote those results that are statistically significant at the 5% level. The map was generated using MATLAB's geoshow routine (MATLAB 2015a, http://www.mathworks.com/products/matlab/).

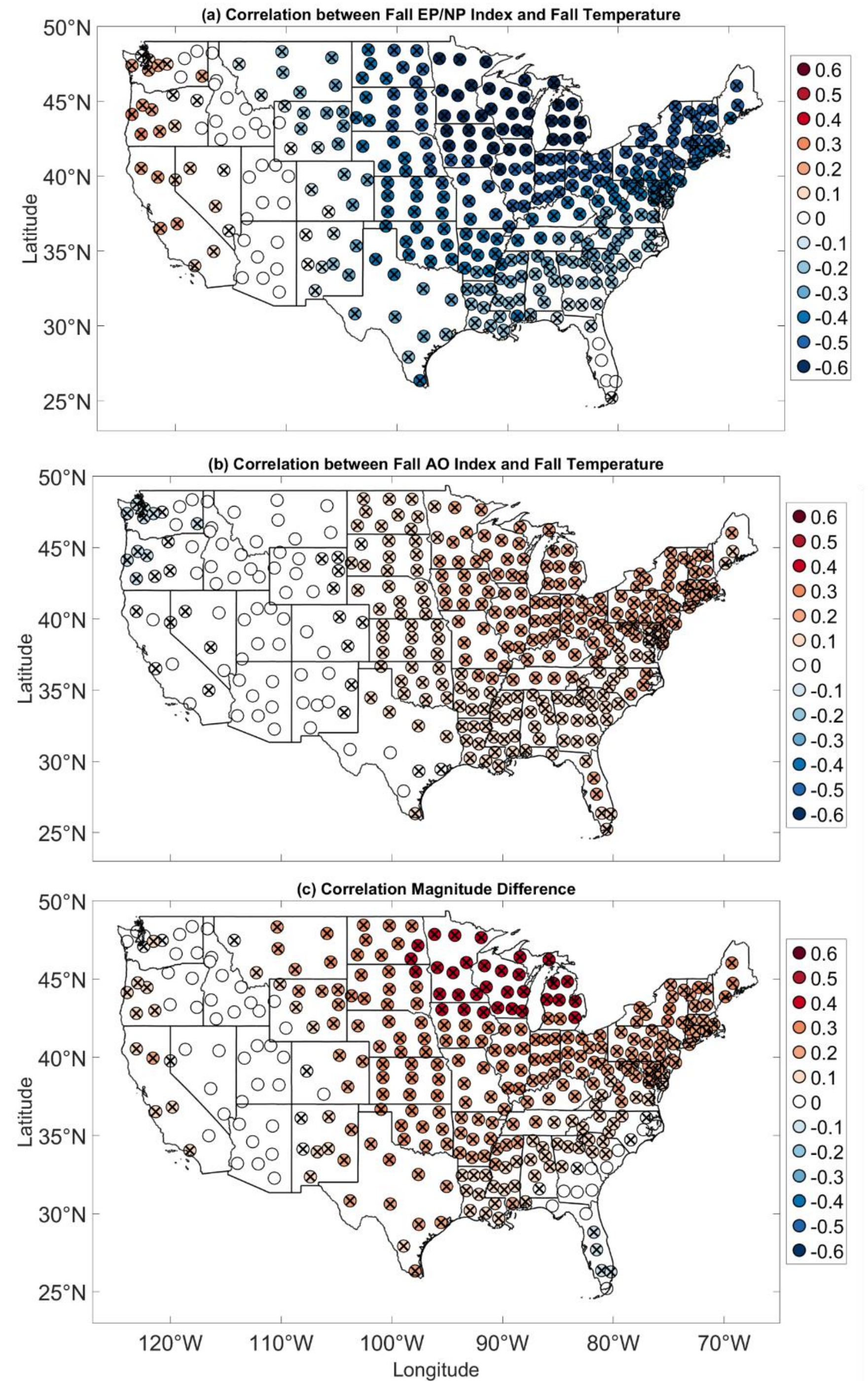

Figure S6. (a) Correlation between Fall EP/NP index and Fall US climate divisional temperature. (b) Same as (a) but for the Fall AO index. (c) Difference between the magnitude of the correlation coefficients in (a) and those in (b). Crosses denote those results that are statistically significant at the 5% level. The map was generated using MATLAB's geoshow routine (MATLAB 2015a, http://www.mathworks.com/products/matlab/).

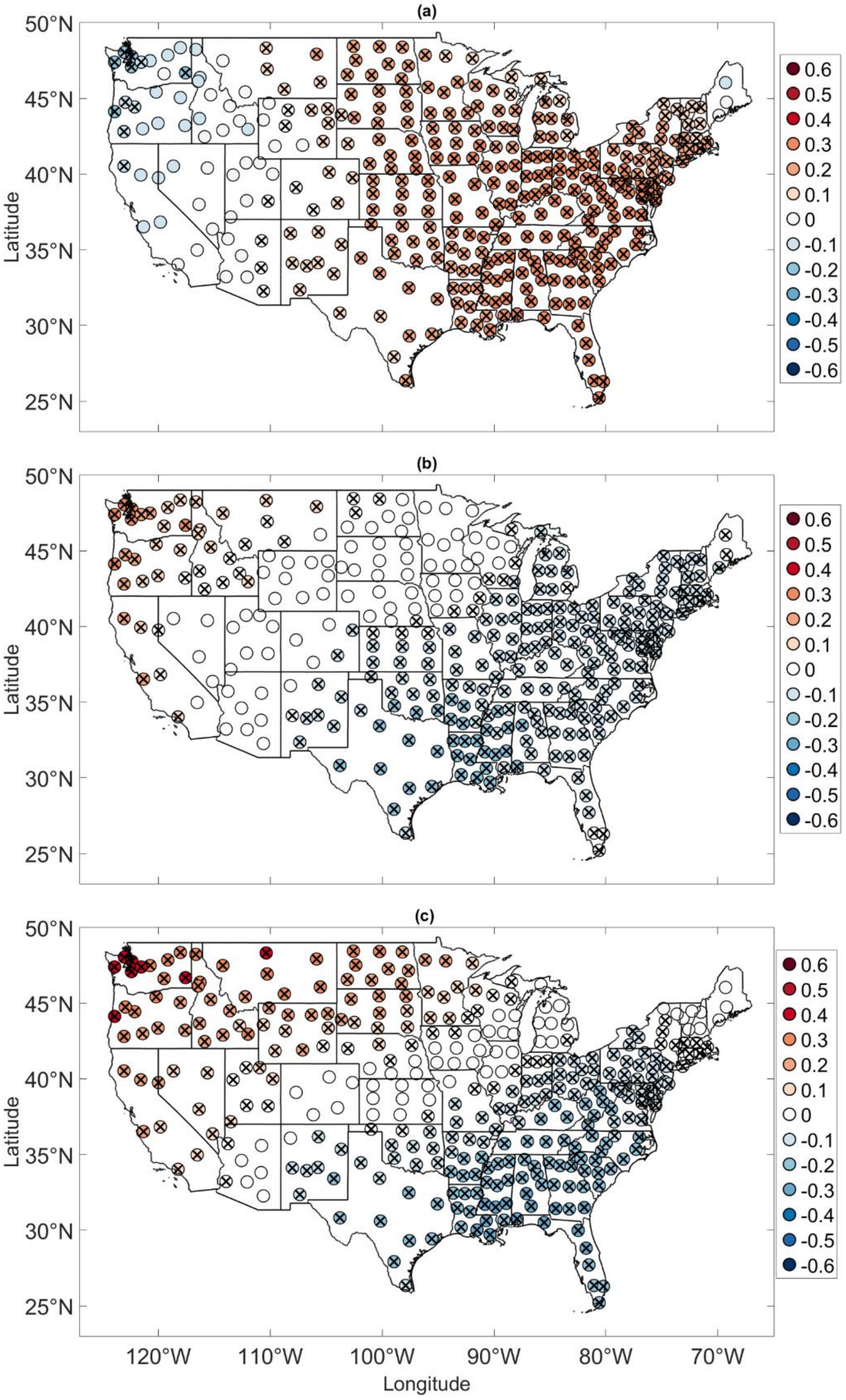

Figure S7. (a) Correlation between the AO index and mean monthly temperature anomalies. Colored dots represent the location and the intensity of the correlation. Crosses denote those correlation coefficients that are statistically significant at the 5% level. (b) Same as (a) but for the PDO index. (c) Same as (b) but for the PNA index. The map was generated using MATLAB's geoshow routine (MATLAB 2015a, http://www.mathworks.com/products/matlab/).

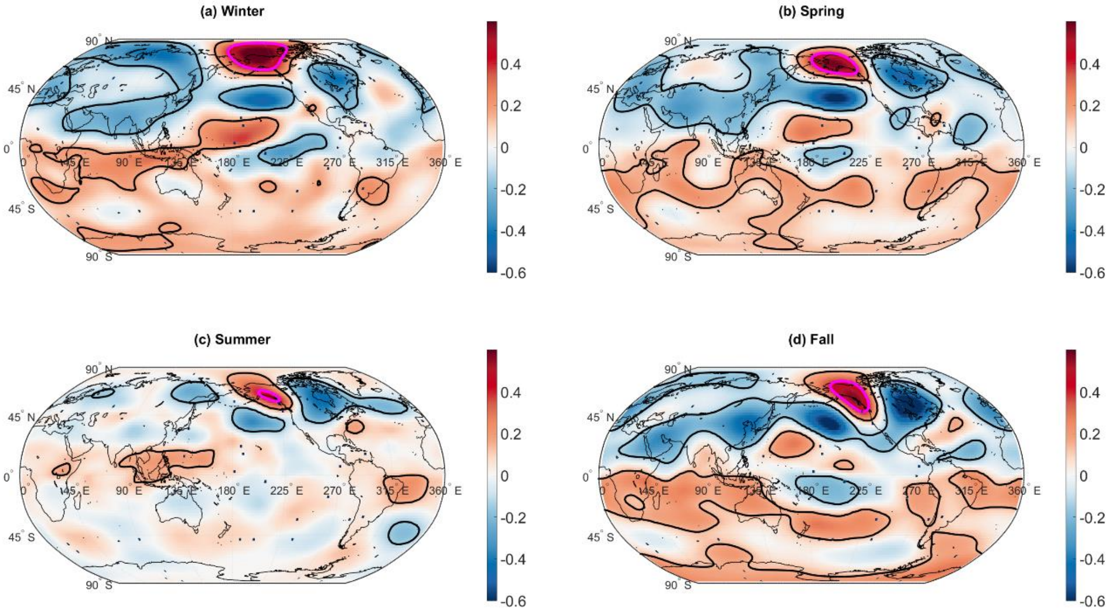

Figure S8. (a) Correlation between winter EP/NP index and winter 300-hPa streamfunction anomalies for the period 1950 to 2015. Contours enclose regions of 5% statistical significance. (b) Same as (a) but for the Spring. (c) Same as (a) but for the summer. (d) Same as (a) but for the fall. The map was generated using MATLAB's geoshow routine (MATLAB 2015a, http://www.mathworks.com/products/matlab/).

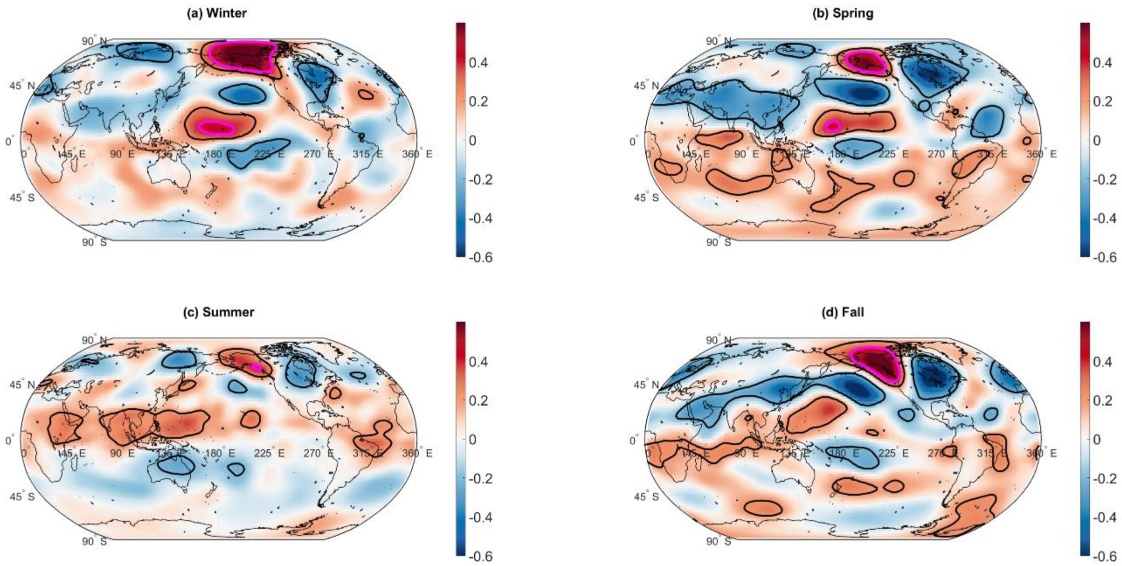

Figure S9. (a) Correlation between winter EP/NP index and winter 300-hPa streamfunction anomalies for the period 1985 to 2015. Contours enclose regions of 5% statistical significance. (b) Same as (a) but for the Spring. (c) Same as (a) but for the summer. (d) Same as (a) but for the fall. The map was generated using MATLAB's geoshow routine (MATLAB 2015a, http://www.mathworks.com/products/matlab/).

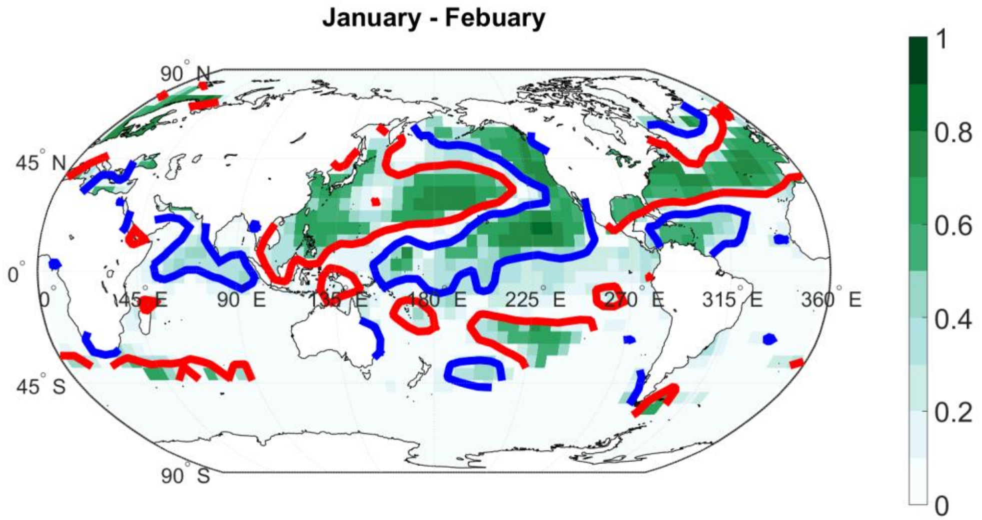

Figure S10. Fraction of US climate divisional for which the temperature time series are statistically correlated with SST. Red contours enclose regions where the mean correlation coefficient exceeds 0.1 and the blue contours enclose regions where the mean correlation coefficient falls below -0.1. Only climate divisions whose temperature time series was negatively correlated with the EP/NP index with at least 5% statistical significance were used in the computation of the means. The map was generated using MATLAB's geoshow routine (MATLAB 2015a, http://www.mathworks.com/products/matlab/).

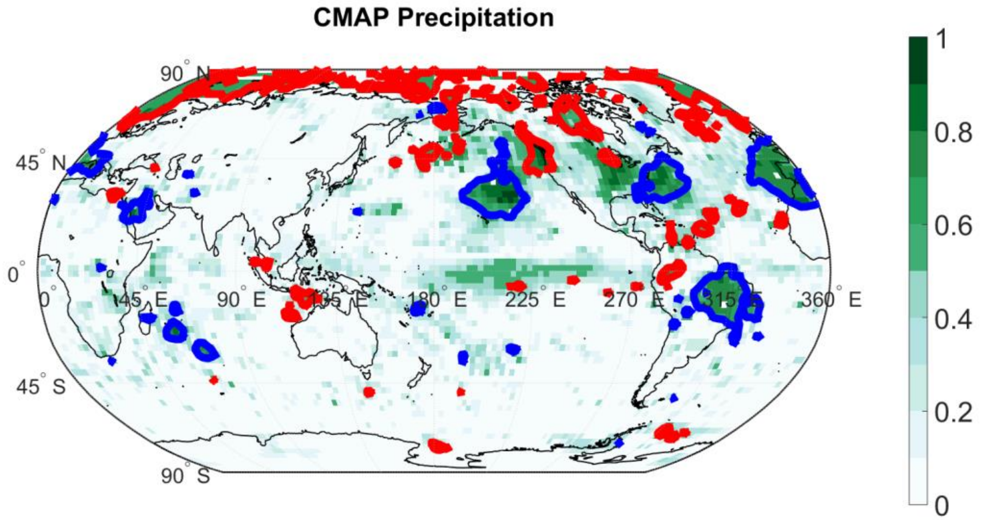

Figure S11. The fraction of statistically significant correlation coefficients calculated between CMAP precipitation and mean monthly temperature anomalies for the 344 US climate divisions from 1980 to 2015. Red contours enclose regions where the mean correlation coefficient exceeds 0.1 and the blue contours enclose regions where the mean correlation coefficient falls below -0.1. Only climate divisions whose temperature time series was negatively correlated with the EP/NP index with at least 5% statistical significance were used in the computation of the means. The map was generated using MATLAB's geoshow routine (MATLAB 2015a, http://www.mathworks.com/products/matlab/).

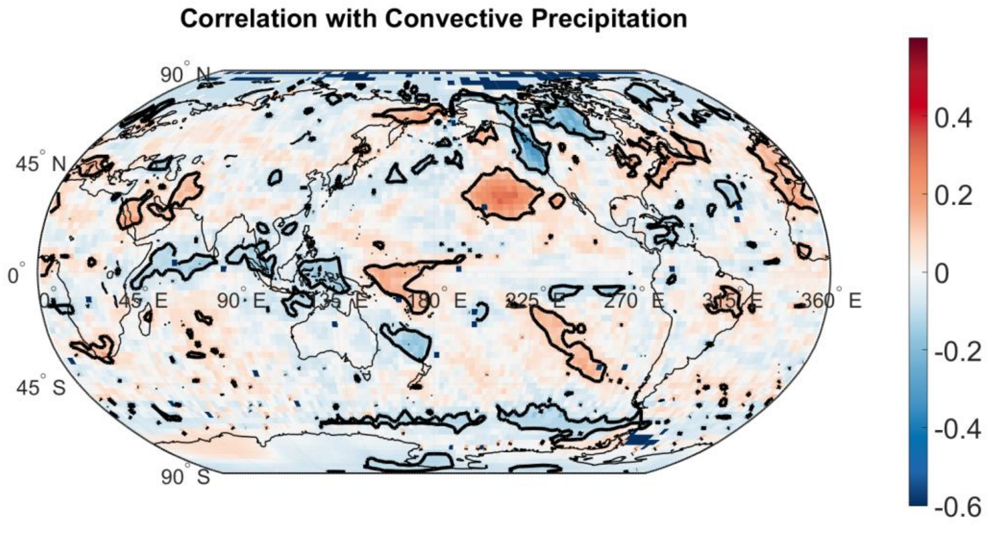

Figure S12. Correlation between precipitation and the EP/NP index from 1980 to 2015. Contours enclose regions of 5% statistical significance. The map was generated using MATLAB's geoshow routine (MATLAB 2015a, http://www.mathworks.com/products/matlab/).

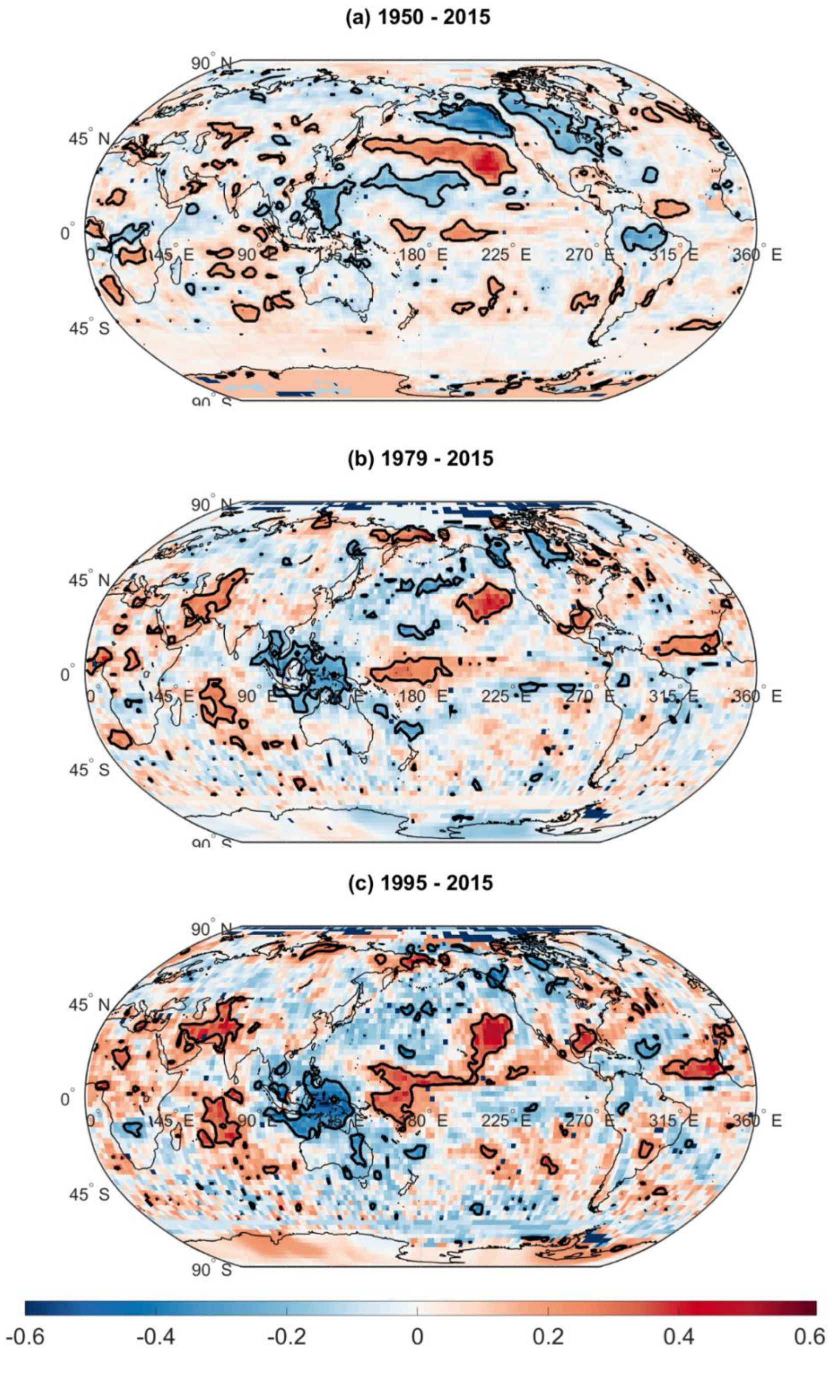

Figure S13. Correlation between the Spring EP/NP index and spring convective precipitation from (a) 1950 to 2015, (b) 1979 – 2015, and (c) from 1995 to 2015. The map was generated using MATLAB's geoshow routine (MATLAB 2015a http://www.mathworks.com/products/matlab/).

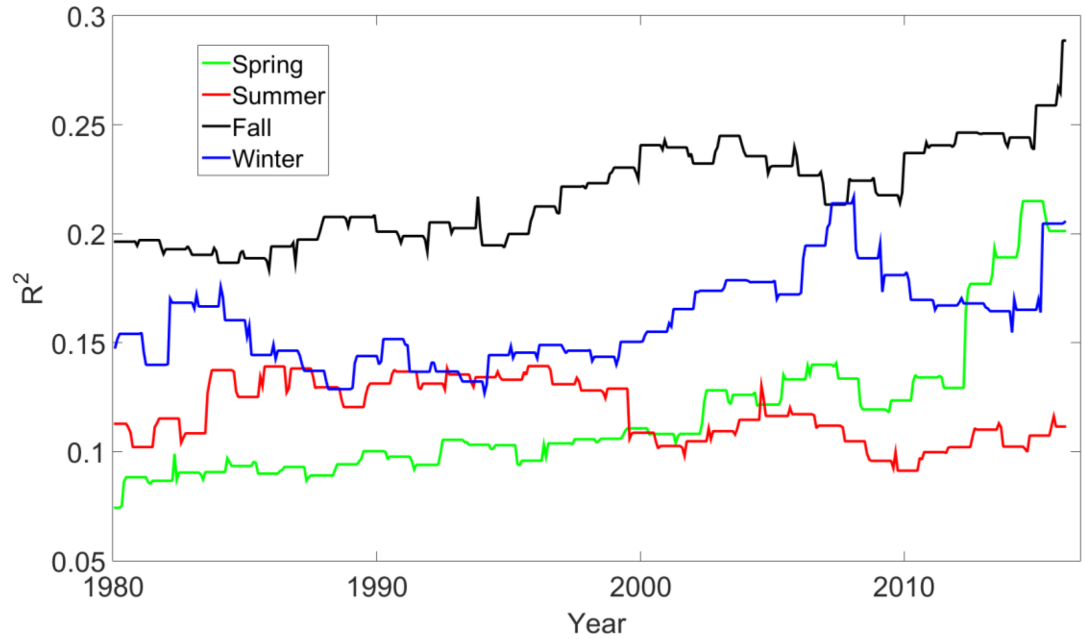

Figure S14. Mean sliding correlation coefficient between the EP/NP index and US climate divisional time series for winter, spring, fall, and summer seasons. Only statistically significant correlation coefficients were used in the computation of the means.

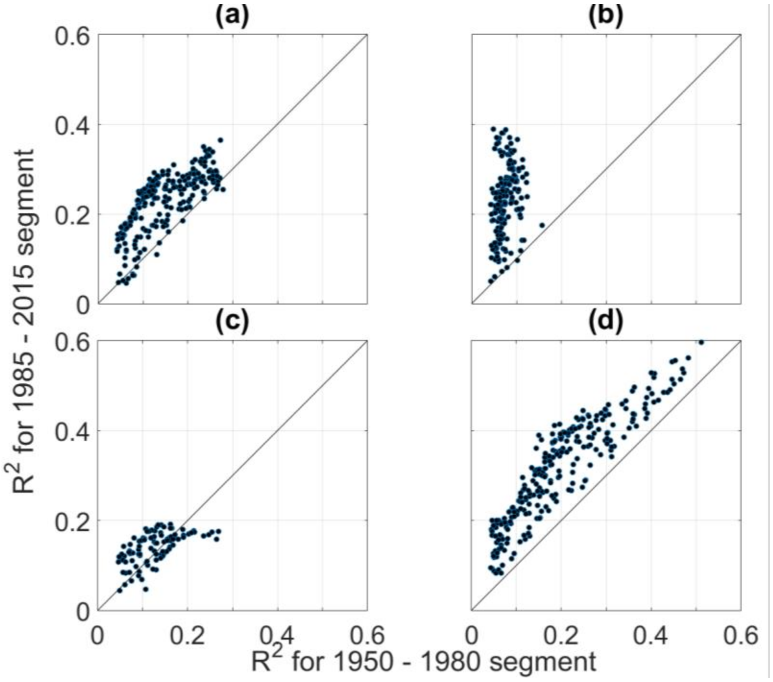

Figure S15. R^2-values in the 1950 – 1980 segment plotted as a function of the R^2-values in the 1985 to 2015 segment for (a) winter, (b) spring, (c) summer, and (d) fall. Points falling on the diagonal line represents those climate divisions for which no difference was found. Only statistically significant R^2-values are plotted.

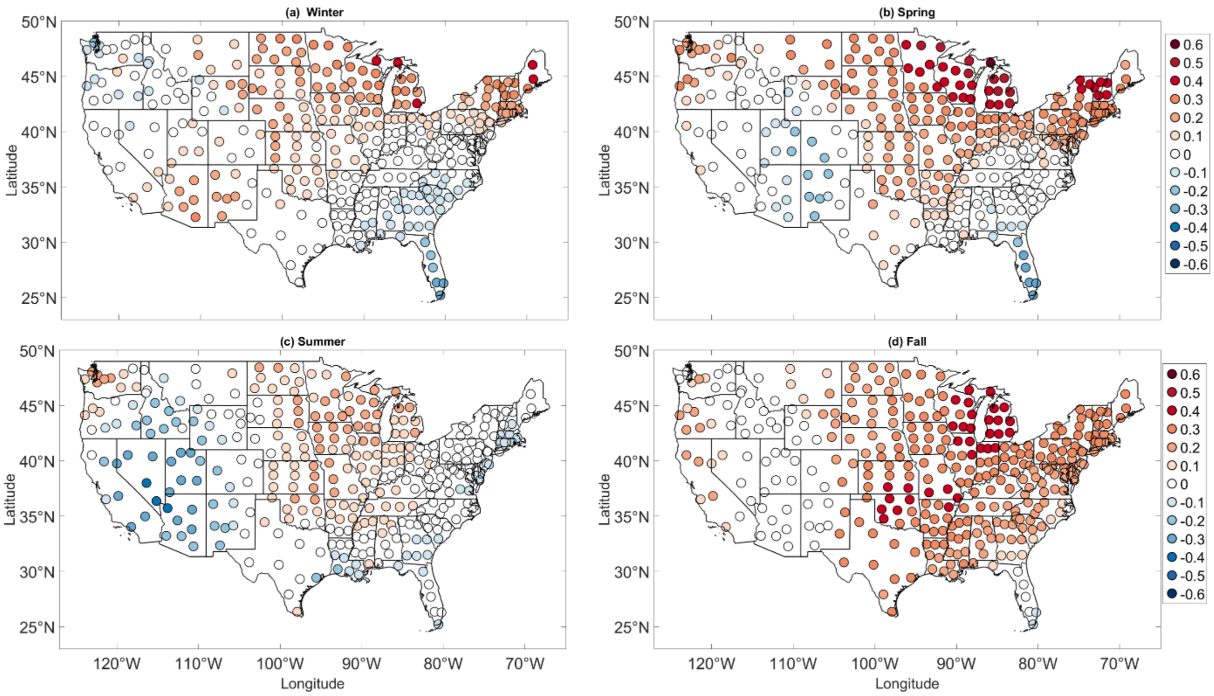

Figure S16. Difference in the magnitude of the correlation coefficients corresponding to the EP/NP-temperature analysis and those associated with the AO-temperature analysis for the (a) winter, (b) spring, (c) summer, and (d) fall from 1985 to 2015. The map was generated using MATLAB's geoshow routine (MATLAB 2015a, http://www.mathworks.com/products/matlab/).

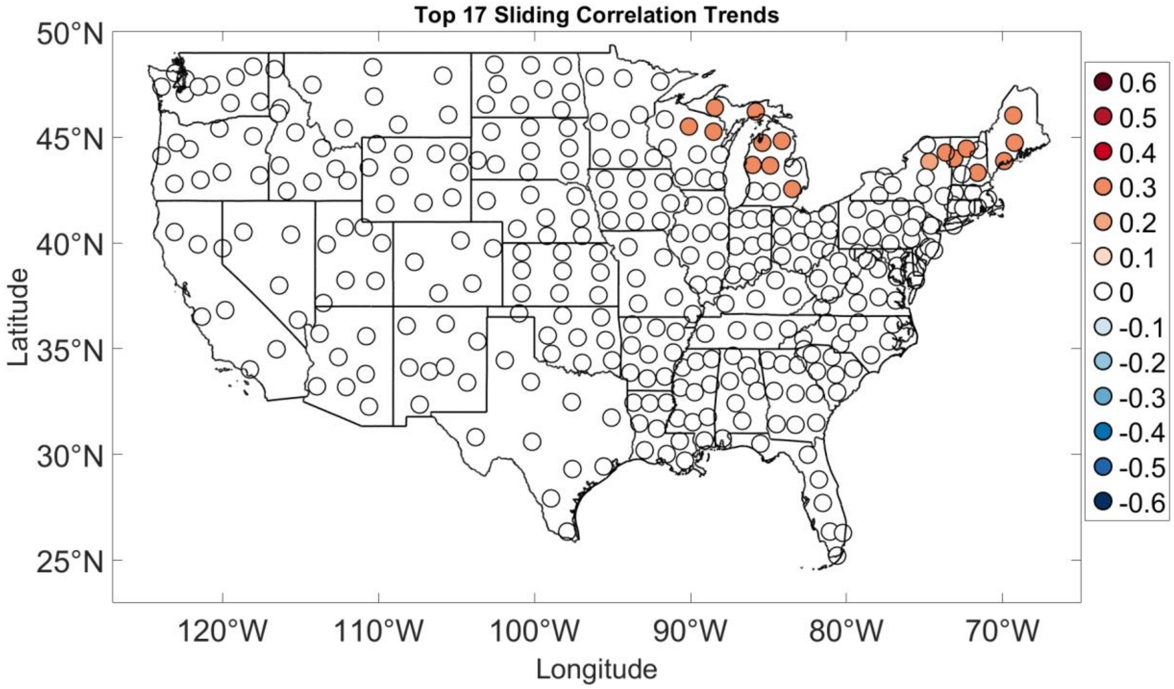

Figure S17. Locations of climate divisions whose R^2 trends are greater than or equal to the 95th percentile of all trends. Values of the trends are also shown. The map was generated using MATLAB's geoshow routine (MATLAB 2015a, http://www.mathworks.com/products/matlab/).

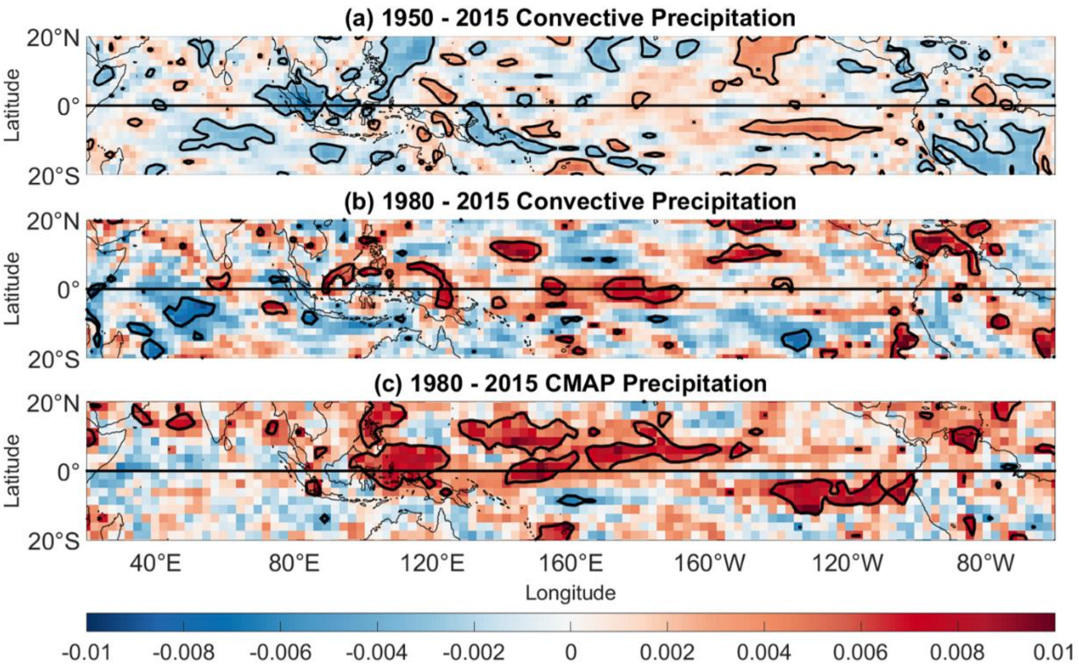

Figure S18**.** (a) Same as Figure 5a for convective precipitation from 1950 to 2015. (b) Same as (a) but for convective precipitation from 1980 to 1950. (c) Same as (b) but for CMAP precipitation from 1980 to 2015. The map was generated using MATLAB's geoshow routine (MATLAB 2015a, http://www.mathworks.com/products/matlab/).

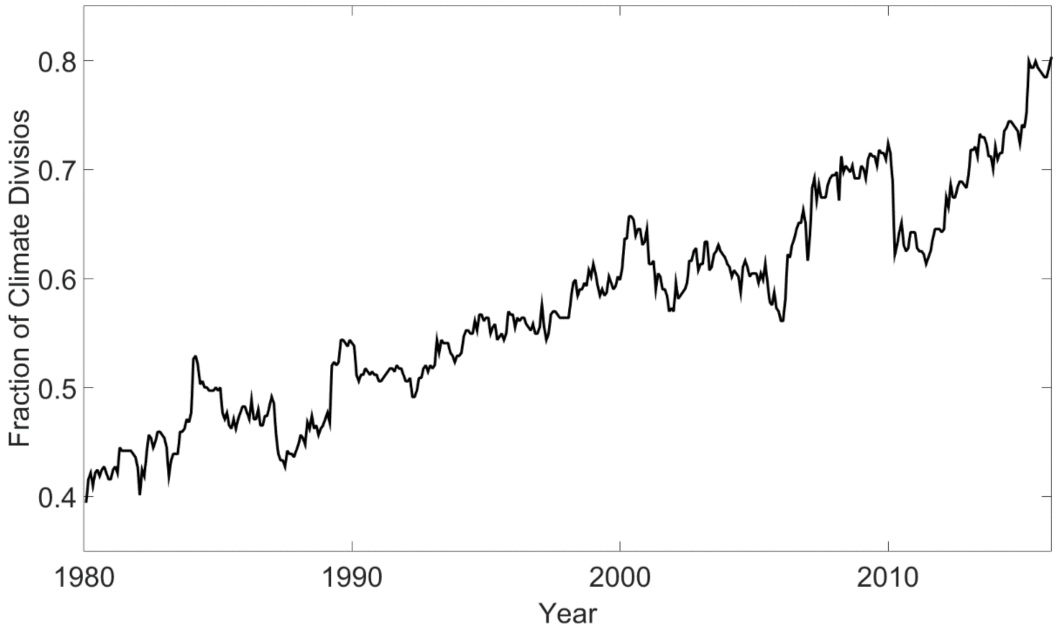

Figure S19. Fraction of US climate divisional time series for which the EP/NP index explained more temperature variability than the AO index. Time series was produced from a 30-year sliding correlation analysis by calculating the number of R^2 values corresponding to the EP/NP index analysis that exceeded that corresponding to the AO index analysis in each 30-yr segment.

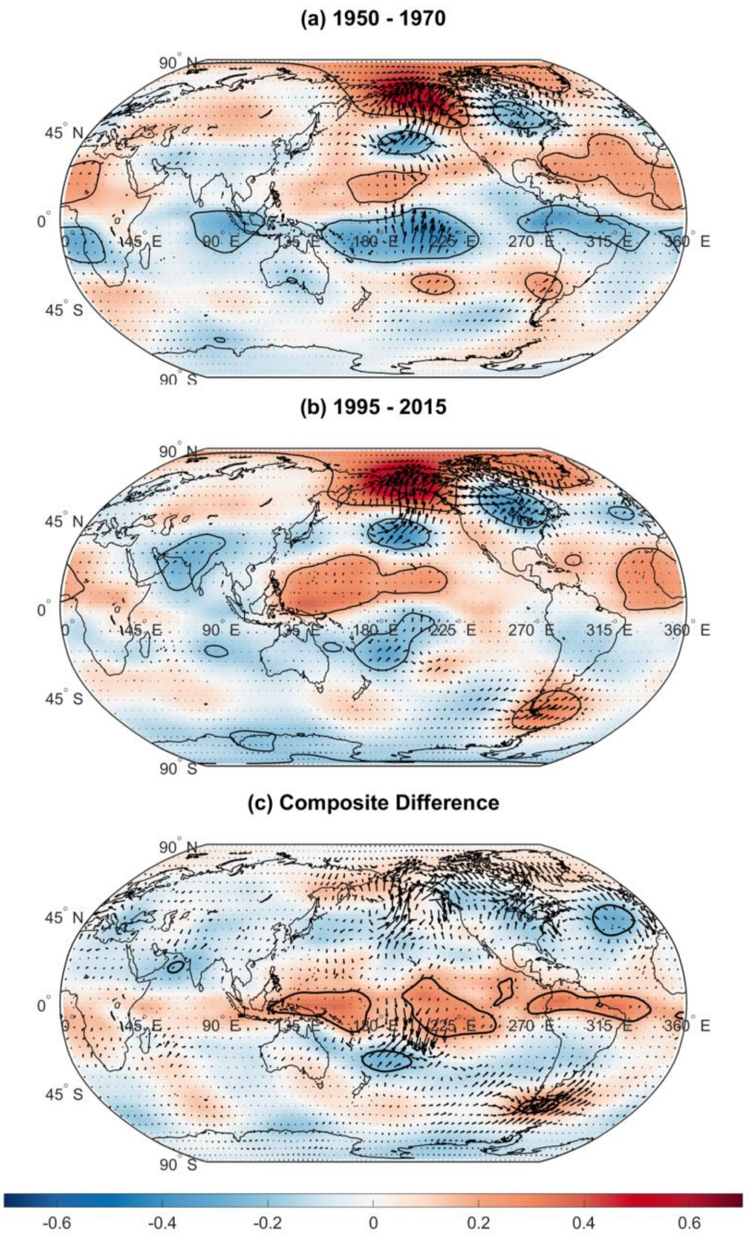

Figure S20. (a) Mean 300-hPa standardized 300-hPa streamfunction anomalies corresponding to the positive EP/NP index for the period 1950 to 1970. (b) Same as (a) but for the period 1995 to 2015. Contours enclose regions where the means are significantly different (at the 5% significance level) from zero, the statistical significance assessed using the student's *t*-test. Arrows indicate the wave activity flux (m^2s^2), which have been scaled relative to the maximum wave activity flux vector length each panel. (c) The difference between the composite means shown in (b) and (a). Wave activity vector lengths are scaled relative to the largest wave activity vector length difference. The map was generated using MATLAB's geoshow routine (MATLAB 2015a, http://www.mathworks.com/products/matlab/)

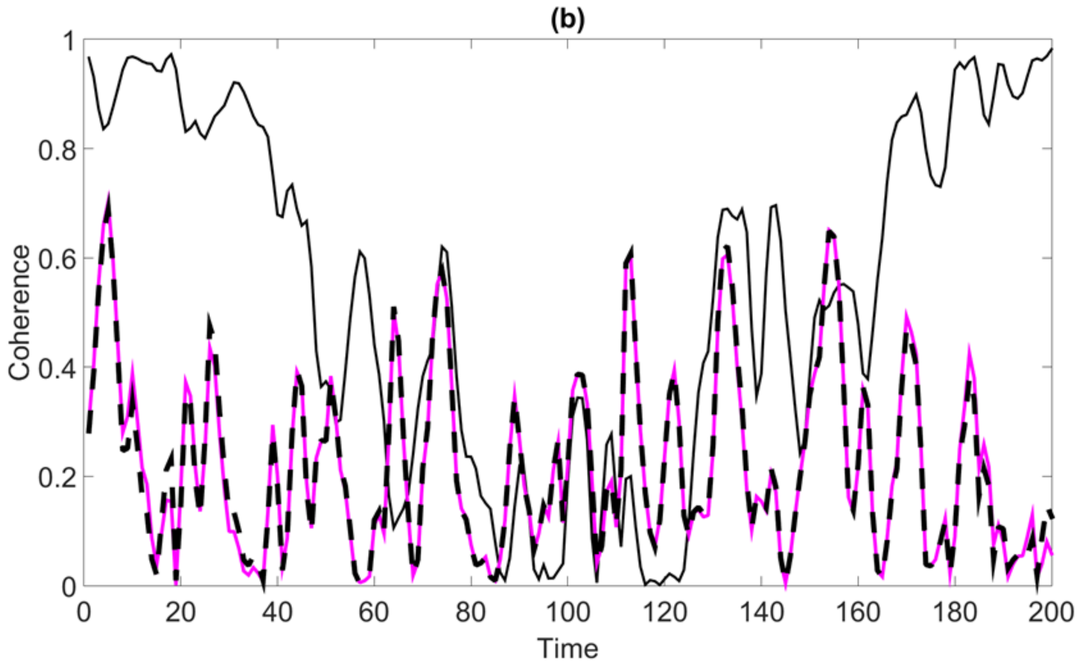

Figure S21. Wavelet scaled coherence between two realizations of a white noise process and between the same realizations with an added linear trend (black curve). The magenta line shows the scale-averaged coherence computed between the white noise realizations before the addition of the trend. The dotted shows the coherence for when the white noise realizations are detrended.

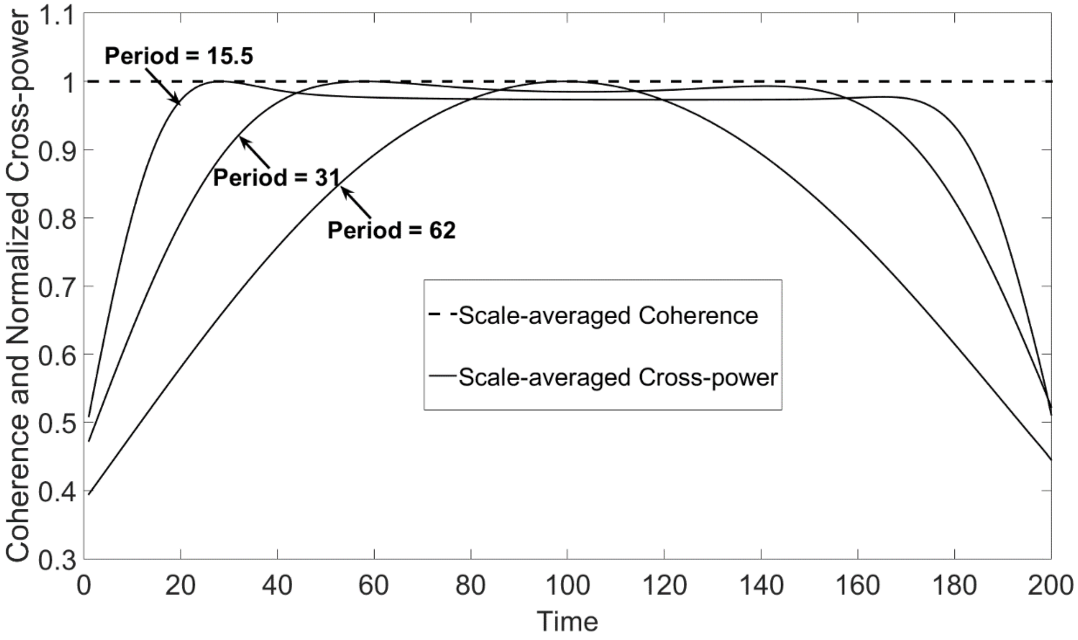

Figure S22. The wavelet cross power between sine waves of length 200 and equal amplitude for three difference cases. In the first case both the sinusoids have period 15.5 and in the second and third cases the sinusoids both have, respectively, periods 31 and 62. Time units are arbitrary. The dotted line is the scale-averaged coherence corresponding to the three cases.
